# Computational analysis and predictive modeling of small molecule modulators of microRNA

**DOI:** 10.1186/1758-2946-4-16

**Published:** 2012-08-13

**Authors:** Salma Jamal, Vinita Periwal, OpenSourceDrugDiscovery Consortium, Vinod Scaria

**Affiliations:** 1 Open Source Drug Discovery Unit, Council of Scientific and Industrial Research (CSIR), Anusandhan Bhavan, 2 Rafi Marg, New Delhi, 110001, India; 2 GN Ramachandran Knowledge Center for Genome Informatics, CSIR Institute of Genomics and Integrative Biology (CSIR-IGIB), Mall Road, Delhi, 110007, India

**Keywords:** microRNA, Machine learning, Maximum common substructure (MCS)

## Abstract

**Background:**

MicroRNAs (miRNA) are small endogenously transcribed regulatory RNA which modulates gene expression at a post transcriptional level. These small RNAs have now been shown to be critical regulators in a number of biological processes in the cell including pathophysiology of diseases like cancers. The increasingly evident roles of microRNA in disease processes have also motivated attempts to target them therapeutically. Recently there has been immense interest in understanding small molecule mediated regulation of RNA, including microRNA.

**Results:**

We have used publicly available datasets of high throughput screens on small molecules with potential to inhibit microRNA. We employed computational methods based on chemical descriptors and machine learning to create predictive computational models for biological activity of small molecules. We further used a substructure based approach to understand common substructures potentially contributing to the activity.

**Conclusion:**

We generated computational models based on Naïve Bayes and Random Forest towards mining small RNA binding molecules from large molecular datasets. We complement this with substructure based approach to identify and understand potentially enriched substructures in the active dataset. We use this approach to identify miRNA binding potential of a set of approved drugs, suggesting a probable novel mechanism of off-target activity of these drugs. To the best of our knowledge, this is the first and most comprehensive computational analysis towards understanding RNA binding activities of small molecules and predictive modeling of these activities.

## Background

MicroRNAs are a well characterized class of small non-coding RNAs now known to be encoded in the genomes of a wide variety of eukaryotes spanning the plant and animal kingdoms of life [1,2]. Recent advancements in the availability of computational and experimental tools have triggered increasing levels of interest to predict and experimentally validate microRNAs and their biological targets and understand their regulatory roles in a wide variety of organisms [3-5]. MicroRNAs typically mediate post-transcriptional regulation of protein-coding genes by binding to the 3’ un-translated regions of the transcripts [6,7]. A number of microRNAs are known to modulate regulation of crucial oncogenes and function both by promoting as well as suppressing oncogenesis and form a distinct class popularly termed as ‘oncomiRs’ [8]. Due to their ubiquitous role in pathological processes, it has been suggested that microRNAs could act as potential drug targets [9-12].

RNA-binding molecules offer an attractive strategy for modulating microRNAs function. The current literature points to a large number of classes of small molecules, including many therapeutically active classes of molecules which have RNA-binding potential [13,14]. In addition a large number of studies have shown potential small-molecules which can bind and modulate non-coding RNA functions [15,16]. Some of the reported molecules like aurintricarboxylic acid, suramin and oxidopamine modulate microRNA processing by inhibiting microRNA loading on the RNA Induced Silencing complex [16], while molecules like enoxacin, a fluoroquinolone antibacterial agent could potentially modulate microRNA biogenesis in a cancer-specific manner [14].

Techniques and assays for screening of small molecules with potential to modulate microRNA function and or action [16] apart from phenotypic or specific expression based screens have been increasingly being adapted for high-throughput screening strategies. The recent advancements in synthesis of compounds and large numbers of new compound libraries currently available for biological screening, poses a high demand for predictive computational methods that can prioritize molecules for biological screening. Previous studies [17,18] have shown the application of Machine Learning in predictive modeling of molecules from high-throughput datasets available in public domain. We have previously used similar strategies using 2D descriptors and activities reported from high-throughput screen data available in public databases like PubChem for prioritization of small molecules with anti-tubercular action based on modeling activities based on concepts of machine learning [19,20]. Apart from Machine learning chemical similarity searching by means of common substructures has been widely used for predicting potential biological activities of compounds and identifying frequently occurring molecular scaffolds in large molecular libraries [21,22].

Here in this manuscript, we describe a computational strategy for predictive modeling of small molecules with potential to inhibit specific microRNAs, based on machine learning from high-throughput screen dataset for modulators of microRNA mir-21 [13], a well studied oncomiR. We show that the methodology is highly accurate with low false positivity. This methodology could be potentially used for computational prioritization of small molecules before performing high-throughput biological assay. We extend our study to analyze common chemical substructures shared between biologically active molecules using a Maximum Common Substructure (MCS) approach. To the best of our knowledge this is the first comprehensive analysis of predictive modeling of small-molecule modulators of microRNA.

## Results and discussion

### Model construction using machine learning algorithms

The bioassay datasets downloaded from PubChem were used to generate 179 2D molecular descriptors using PowerMV. Data processing (described in Materials and Methods) resulted in 154 molecular descriptors (Additional file 1). The training file was loaded in Weka for classification tasks. Owing to the large size of the dataset Weka was started with an increased heap space of 4 GB to handle out-of-memory exception. Initially standard classifiers were used to generate the models, however, due to the low true positives rate, cost sensitivity was introduced and the cost was incremented so as to stay around the upper limit of false positives (i.e. 20%). Final misclassification cost of false negatives used for both the classifiers is given in Table 1. The Naive Bayes required a lower misclassification cost and was very quick in building the model. A number of models were trained with different misclassification cost settings. The best models from both classifiers were selected based on their performance as evaluated by different statistical measures (Table 1).

**Table 1:** Classification results

Classifier*	**Cost**	**TP rate**	**FP Rate**	**ROC area**	**Accuracy (%)**	**BCR**^**#**^**(%)**
CSC Naïve Bayes	38	54.5	20.1	72.8	79.85	66
CSC Random Forest	65000	60.2	19.0	77.3	81.19	70

*CSC denotes *CostSensitiveClassifier,* # Balanced Classification Rate.

### Evaluation of models

**Figure 1:**
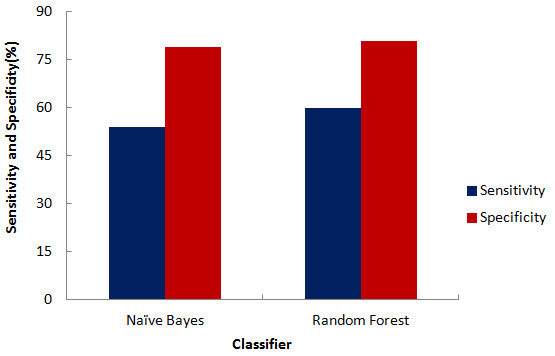
Plot of sensitivity and specificity.

Initial evaluation was performed using sensitivity and specificity plots (Figure 1) for best models of both the classifiers. An experiment generating high sensitivity and specificity is considered to have low error rates. As can be visualized from the graph, though Random Forest is more sensitive as compared to Naïve Bayes, both the classifiers are equally specific in their predictions. Traditionally, the most simple and commonly used assessment metric for describing the overall effectiveness of a classifier was by its accuracy. In the present study both the classifiers produced an impressive accuracy of nearly 80%, but this measure has its own short-comings when applied to highly imbalanced datasets where positive examples are under-represented as compared to negative examples as in our dataset. In lieu of this, other performance measures are now being widely adopted so as to provide a more detailed and comprehensive evaluation of the datasets having a class imbalance problem.

**Figure 2:**
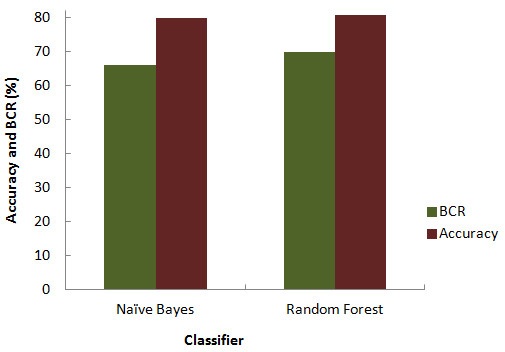
Comparison of accuracy and balanced classification rate.

**Figure 3:**
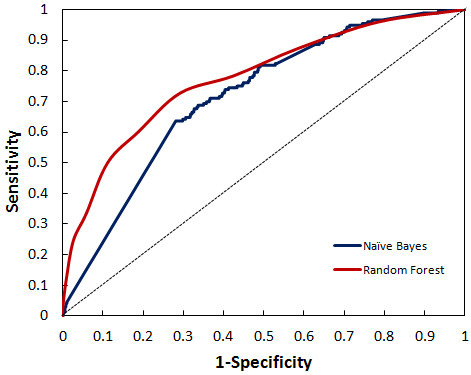
ROC plot depicting significant AUC curve values for random forest and naïve bayes.

BCR is a popularly used assessment metrics for imbalanced datasets. Since BCR provides an average of sensitivity and specificity, it gives a more precise picture of classifier effectiveness. Balanced Accuracy of the classifiers also turned out to be as good as was accuracy alone (Figure 2). BCR value of Random Forest and Naïve Bayes was 70% and 66% respectively. Relative classifier performance can be easily compared by ROC curve analysis. It is extremely efficient measure as it provides visualization of relative trade-offs between true positives and false positives. The Area under the curve (AUC) obtained from ROC plot of the two classifiers depicted in Figure 3, suggested that Random Forest performed better producing a significant AUC of 77.3% compared to Naïve Bayes. A completely random guess by the classifier would have resulted in points lying along the diagonal dividing the ROC space.

### Evaluation of enriched substructures

**Figure 4:**
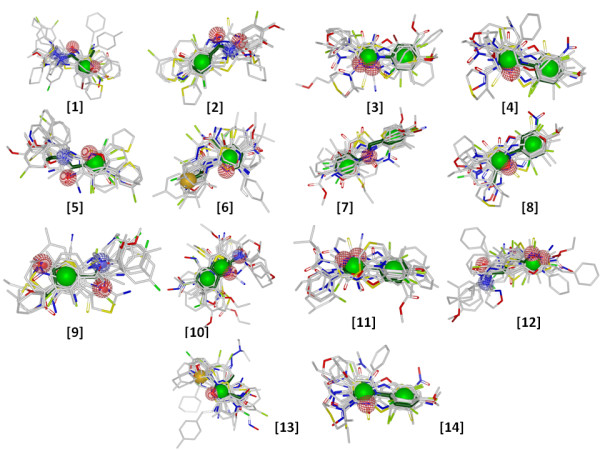
**Molecular overlay.** Alignment of 14 enriched scaffolds (dark green) with top 20 compounds of active dataset. Ranking was obtained from their Tanimoto similarity and overlap with the reference scaffold.

Although molecular descriptor based methods are computationally simple and effective in practice but they share several shortcomings most important being the inability to identify local similarity between structures. This is important for chemists in understanding and synthesizing molecules based on active scaffolds. The active dataset containing 883 compounds was clustered using the LibMCS algorithm which generated a total of 1151 hierarchical scaffolds/substructures spanning up to 6 levels. Only top level clusters were selected for further analysis. The number of clusters at level 6 was 182. Out of the 182 clusters, 71 were singletons which were removed from further analysis whereas remaining selected 111 clusters had compounds count ranging from 2–144. The number of occurrence of each of the 111 substructures in the actives and the inactives dataset was determined. We considered only substructures with a frequency of occurrence of > 1% in the active dataset which accounted for 41 scaffolds. The enrichment and its significance, was analyzed by chi-square test (Table 2). Analysis revealed 14 significantly enriched scaffolds in the active dataset which had p-value less than 0.01 and an enrichment factor > 2. We also performed an alignment of the 14 enriched scaffolds with top 20 compounds of the active dataset (Figure 4). The Tanimoto similarity and overlap between query scaffold and target active dataset were used as a means to rank matches.

**Table 2:** Significantly enriched scaffolds in the active dataset

**Scaffold_No.**	**Scaffold_structure**	**Actives**	**Inactives**	**Chi-square**	**p-value**	**Enrichment factor**
Scaffold 1		19	86	1144.377	0.00	75.49
Scaffold 2		14	97	579.406	0.00	49.32
Scaffold 3		15	623	93.197	4.73E-22	8.22
		13	628	66.565	3.38E-16	7.07
		14	692	69.573	7.36E-17	6.91
		14	878	50.204	1.39E-12	5.44
		28	2186	72.564	1.62E-17	4.37
		14	1140	33.804	6.09E-09	4.19
		12	1090	24.158	8.87E-07	3.76
Scaffold 10		21	1999	39.091	4.04E-10	3.58
Scaffold 11		14	1356	25.217	5.12E-07	3.52
Scaffold 12		10	1086	14.563	1.35E-04	3.14
Scaffold 13		11	1405	11.505	6.94E-04	2.67
Scaffold 14		14	1852	13.566	2.30E-04	2.58

### DrugBank and Protein Data Bank (PDB) database screening

We used the predictive models to screen approved drugs from DrugBank database [23]. Out of the 1410 approved drugs NB model predicted 205 drugs and RF model predicted 74 drugs to be active against miR-21 (Additional file 2). A consensus from both the models resulted in 43 drugs. A clustering analysis of the 43 drugs (Additional file 3) revealed the presence of mostly heterocyclic compounds comprising benzenes, quinolines, furans, pyridines and their derivatives. The 14 significantly enriched scaffolds were searched in the Protein Data Bank [24] to identify any similarity with known RNA binding ligands. One positive hit was obtained (Additional file 4) for Scaffold 3 which matched with the ligand ‘triazole-acridine’ (PDB-id: R14) which is known to bind to telomeric RNA-quadruplex (PDB-id: 3MIJ) [25].

### Virtual screening of experimentally identified novel miRNA inhibitors

We have also used the predicted models to screen a set of novel molecules identified as miRNA inhibitors derived from different literature sources [14-16,26,27]. Out of the 37 molecules reported as actives in these literatures, NB predicted 12 molecules as actives and RF predicted 11 molecules as actives (Additional file 5). Consensus predictions made by both the models suggested 11 molecules to have probable activity against miR-21.

## Conclusion

Understanding small molecules that bind to RNA could have implications both in modulating RNA levels for research as well as therapeutic applications. In this study, we have been successful in creating predictive computational models for small molecules with potential to bind and inhibit microRNA action using machine learning algorithms and chemical descriptors. We show the methodology is highly accurate with low false positivity. This methodology could be potentially used for computational screen of datasets before performing high-throughput screen as well as picking potential hits from large chemical structure datasets. In addition we have evaluated the maximally enriched substructures in the active dataset of small molecules with activity against mir-21. Apart from being involved in the pathogenesis of neoplasia, mir-21 is also known to be involved in the pathogenesis of Mycobacterium leprae [28] and is suggested to be involved in the modulation of immune responses in intracellular pathogens including Mycobacterium tuberculosis [29]. Recent evidence has also suggested that microRNA apart from others to be differentially expressed in individuals with latent tuberculosis [30].This would also serve as the starting point to understand and design molecule libraries both virtual as well as experimental for specific activities for both research and therapeutic applications. To the best of our knowledge this is the first comprehensive analysis of predictive modeling of small-molecule modulators of microRNA.

## Methods

### Data source

The dataset [AID: 2289] consisting of modulators of human microRNA, miR-21 was downloaded from PubChem [31]. The high-throughput screen consisted of a total of 3,33,521 tested compounds. Compounds were characterized based on a compound ranking system called ‘PubChem Activity Score’. Compounds having an activity score between 40 and 100 were considered as active (3282), all compounds with a score of 0 were inactives (3,01,747) and the ones having a score between 1 and 39 were labeled as inconclusive (28,713). The active and inactive sets were downloaded in Structure Data Format (SDF).

The bioactivity of compounds in the high throughput screen of PubChem AID2289 has been measured in a cell-based Firefly Luciferase (FLuc) reporter gene assay. However, it has earlier been reported [32,33] that compounds that resemble substrates of FLuc can potentially function as competitive inhibitors of the enzyme thereby resulting in counterintuitive phenomenon of signal activation. The apparent increase in luminescence could thus be mistakenly interpreted as an activity. Therefore, we also used the counter-screen of mir-21 project (AID: 588342) that uses a ~350 k library of MLSMR compounds to filter out true positives from potentially false positives. The overlapping revealed that 2399 compounds in the active set of AID2289 are inhibitors of FLuc rather than our target miR-21. All overlaps were filtered out and only 883 true positives were considered as actives for modeling experiments (Additional file 6).

### Dataset preparation

The chemical structures downloaded from PubChem were imported and 2D descriptors were generated using PowerMV [34]. The large dataset was split into smaller files using SplitSDFiles from Mayachem tools [35]. A total of 179 descriptors were calculated which includes 147 pharmacophore fingerprints, 24 weighted burden number and 8 property descriptors (Additional file 1). For the bit string descriptors, each bit was set to ‘1’ when a certain feature was presented and ‘0’ when it was not. The attributes having bit string descriptor values of only one value throughout the dataset (all 0’s or all 1’s) were filtered. The dataset was split into 20% test set and the 80% training-cum-validation set to build the model.

### Cost sensitive classification

One of the caveats with the virtual screening of bioassay data is the imbalance between active and inactive compounds [36]. A dataset is considered imbalanced when one class is represented by large number of entities as compared to other. To overcome this problem cost-sensitive classification has been used previously [37]. In cost sensitive learning, misclassification of the marginal class is assigned a high cost which the algorithm then attempts to lessen. We used Weka (Waikato Environment for Knowledge Analysis), a popular suite of machine learning software, to perform modeling tasks [38]. In Weka, cost sensitivity is introduced by means of a confusion matrix. In the present binary classification scheme a 2x2 matrix was deployed to predict the class with the minimum expected misclassification cost setting. A 2x2 confusion matrix consists of four sections: True positives (TP) for active compounds correctly classified as active, false positives (FP) for inactive compounds incorrectly classified as active, true negatives (TN) for inactive compounds correctly classified as inactive and false negatives (FN) for active compounds incorrectly classified as inactive. As false negatives are deemed to be more important in any experiment, misclassification cost was set for false negatives and was incremented serially so as to optimize the predictions. The maximum false positive rate is constrained to approximately 20%. The optimal misclassification cost setting for each classifier in the Weka cost matrix depends on the base classifier used. The model was first build with training dataset and 5-fold cross validation was used during training of data. Cross validation is a technique in which data is partitioned into subsets, performing the analysis on one subset (called the training set), and validating the analysis on the other subset (called the validation set or testing set). The base classifiers used were Naive Bayes and Random forest. For both Naive Bayes and Random forest, cost sensitivity was employed.

### Classification methods

Machine learning is a field of artificial intelligence and is based on prediction of a set of outcomes, based on known properties learned from a dataset of known outcomes, otherwise termed as the training data. In our experiment the following algorithms were used which can be formulated in terms of machine learning methods.

*Naïve Bayes* is one of the simplest probabilistic classifier. The technique is based on Bayes theorem in statistics. A Bayesian classifier considers each structural feature or descriptor independent of the other descriptors, and the probability of activity is considered to be proportional to the ratio of actives to inactives that share the descriptor value. The final probability that a compound is active is a product of all descriptor based probabilities [39].

*Random Forest* was first described by [Leo Breiman 40]. It is an ensemble classifier methodology based on decision trees. The algorithm tries to find as good a distinction as possible between active compounds and others, on the basis of a set of molecular descriptors. It identifies features shared by different subsets of active compounds and accordingly filters out compounds within the target data set in which these combinations are lacking. It is the most accurate classifiers available.

### Model evaluation

We used various statistical measures such as Accuracy, Sensitivity, Specificity, Balanced Classification Rate (BCR) and Receiver Operating Characteristic (ROC) to evaluate the models. Sensitivity, Specificity and Accuracy are expressed in terms of true positive (TP), false negative (FN), true negative (TN), false positive (FP) rates. A True Positive Rate (TPR) is the proportion of actual positives which are correctly predicted as actives (TP/TP + FN). False Positive Rate (FPR) is ratio of predicted false actives to actual number of inactives (FP/FP + TN). Accuracy indicates overall effectiveness of the classifier. It can be calculated as (TP + TN/TP + TN + FP + FN). Sensitivity refers to proportion of actual positives which are predicted positives (TP/TP + FN). Specificity refers to proportion of actual negatives which are predicted negatives (TN/TN + FP). Balanced Classification Rate (BCR) is the average of sensitivity and specificity which may be defined as a measure to test classifiers ability to avoid false classification.

### Maximum common substructure search

A maximum common substructure (MCS) based approach was used to identify potentially enriched bioactive molecules. We used the hierarchical clustering algorithm ‘LibMCS’, available from [ChemAxon 41] to recognize the substructure common to a pair of molecules. This MCS based classification of molecules creates disjoint subsets, where one molecule belongs to one cluster only. The size of the MCS is determined as a function of the numbers of the constituent atoms which was empirically set to a threshold of ”10 atoms” in this study owing to the complexity of the structures involved and computation required to generate the clusters.

The molecular scaffolds generated as a result of clustering were thus used as SMILES query to search for substructures in both active and inactive target datasets. This was accomplished using the ‘*jcsearch*’ algorithm available from [ChemAxon 42]. The substructures were later evaluated for enrichment using chi-square test. The p-values were used to evaluate the significance of enrichment. We used substructures which have at least > 1% matches among the active dataset entries. We also calculated enrichment factor and used an empirical threshold of 2 to prioritize molecules for further analysis. A molecular alignment of the selected scaffolds with molecules of active dataset was performed using the vROCS (release 3.1.2) [43] and visualized in VIDA (4.1.1) [44] available from OpenEye Scientific Software, Inc. [45].

## Competing interests

The authors declare that they have no competing interests.

## Authors’ contribution

SJ and VP under the guidance of VS designed the study, carried out the work flow and performed the analysis. OSDDC was involved in regular discussions and supported the work. All authors contributed to manuscript writing, and have read and approved, the final manuscript.

## Supplementary Material

Additional file 1 List of descriptors calculated and filtered for AID2289 dataset. Click here for file

Additional file 2 DrugBank predictions of NB and RF models. Click here for file

Additional file 3 Depicting clustering of 43 drugs from DrugBank predicted actives against miR-21, based on Tanimoto similarity. Click here for file

Additional file 4 Scaffold hits from PDB. Click here for file

Additional file 5 NB and RF model predictions on 37 novel small molecule miRNA inhibitors reported in various literatures. Click here for file

Additional file 6 Overlap between miR-21 assay (AID2289) and FLuc inhibitors. Click here for file
