# Supplementary figures and images for: Computational analysis and predictive modeling of small molecule modulators of microRNA
Source: J Cheminform. 2012 Aug 13;4:16. doi: 10.1186/1758-2946-4-16 (PMC3466443; doi:10.1186/1758-2946-4-16)

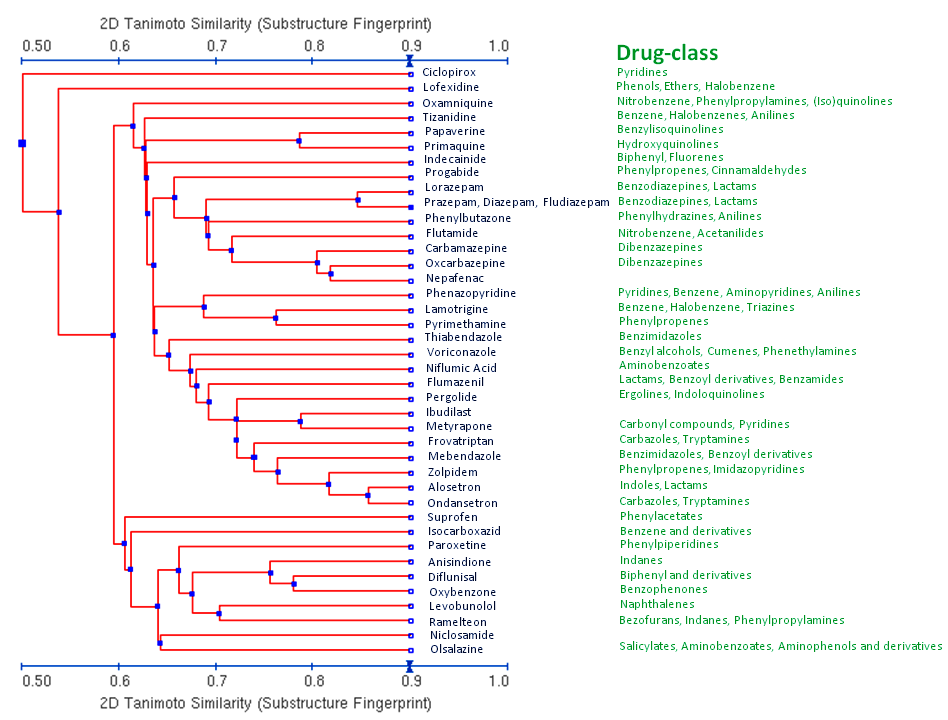

Supplement: Additional file 3 — Depicting clustering of 43 drugs from DrugBank predicted actives against miR-21, based on Tanimoto similarity. [file 1758-2946-4-16-S3.tiff]

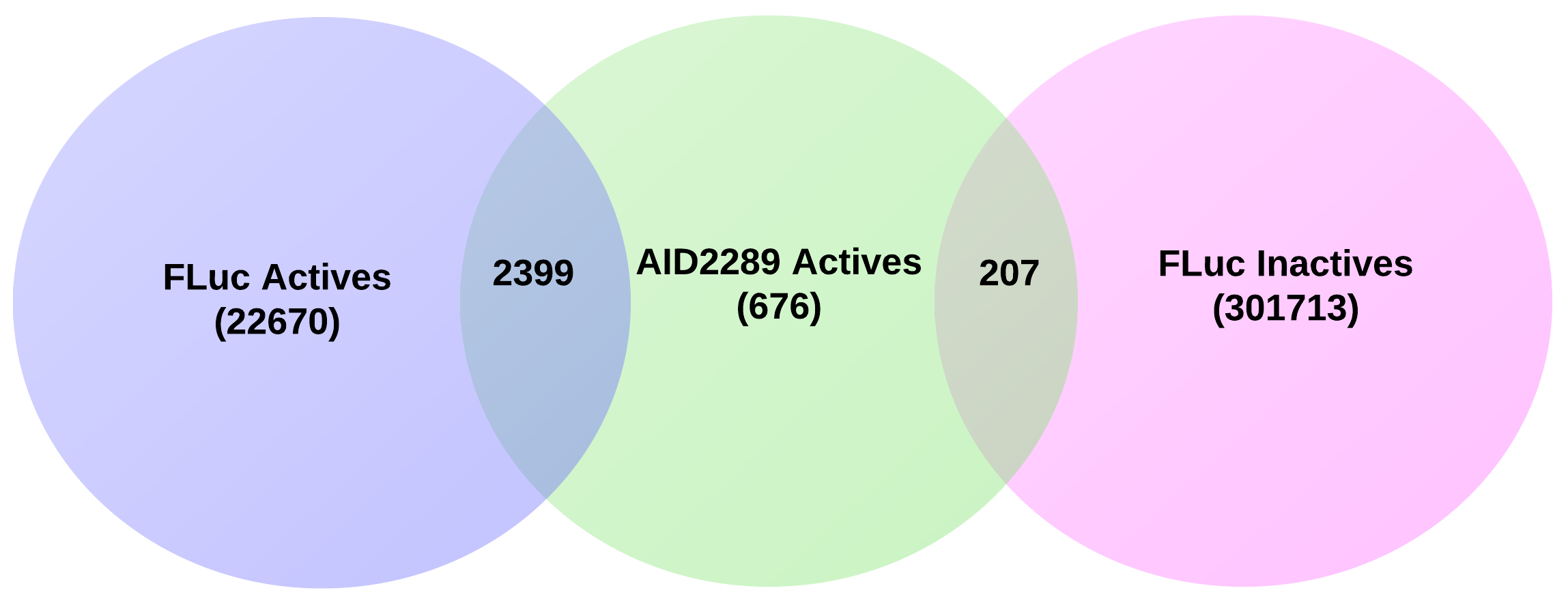

Supplement: Additional file 6 — Overlap between miR-21 assay (AID2289) and FLuc inhibitors. [file 1758-2946-4-16-S6.png]
