# Supplementary material for: Computational analysis and predictive modeling of small molecule modulators of microRNA
Source: J Cheminform. 2012 Aug 13;4:16. doi: 10.1186/1758-2946-4-16 (PMC3466443; doi:10.1186/1758-2946-4-16)
Supplement: Additional file 4 — Scaffold hits from PDB. [file 1758-2946-4-16-S4.doc]

**Additional file 5** DOC file containing scaffold hits from PDB

| **Scaffold** | **PDB_Ligand** | **PDB_structure** |
| --- | --- | --- |
| 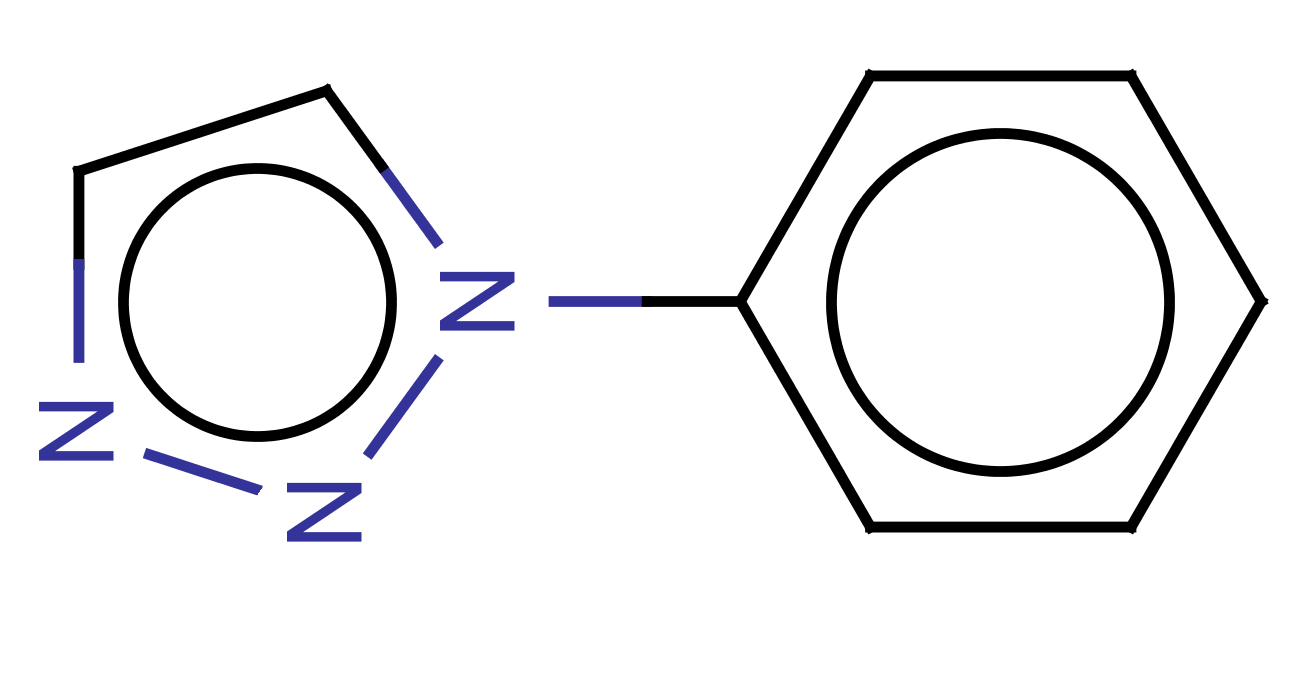 | 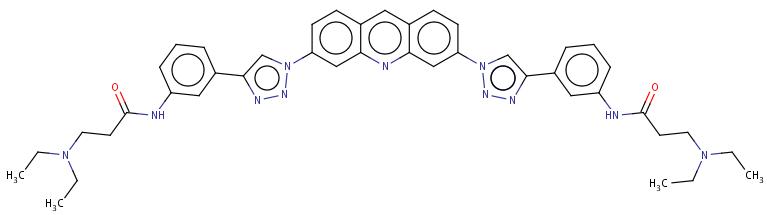 | 3MIJ |
