# Supplementary material for: Computational analysis and predictive modeling of small molecule modulators of microRNA
Source: J Cheminform. 2012 Aug 13;4:16. doi: 10.1186/1758-2946-4-16 (PMC3466443; doi:10.1186/1758-2946-4-16)
Supplement: Additional file 5 — NB and RF model predictions on 37 novel small molecule miRNA inhibitors reported in various literatures. [file 1758-2946-4-16-S5.doc]

**Additional file 6** Virtual screening of experimentally identified novel miRNA inhibitors. Using a consensus of predictions made by NB and RF, 11 compounds marked in bold are predicted to be active against miR-21.

| **Compound_ID** | **Structure** | **Target** | **NB_Predictions** | **RF_predictions** |
| --- | --- | --- | --- | --- |
| **1a [1]** | **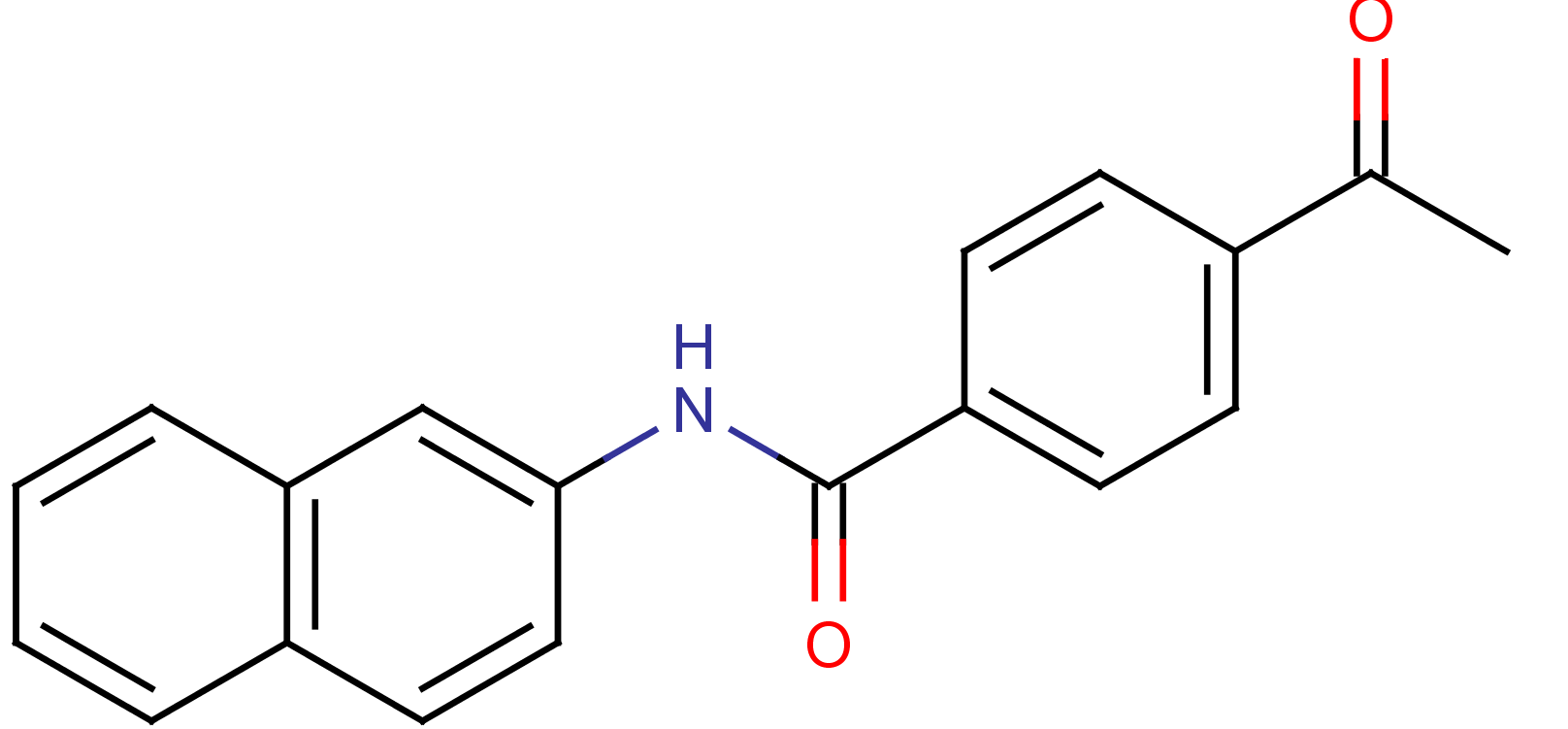** | **mir122** | **Active** | **Active** |
| **1b [1]** | **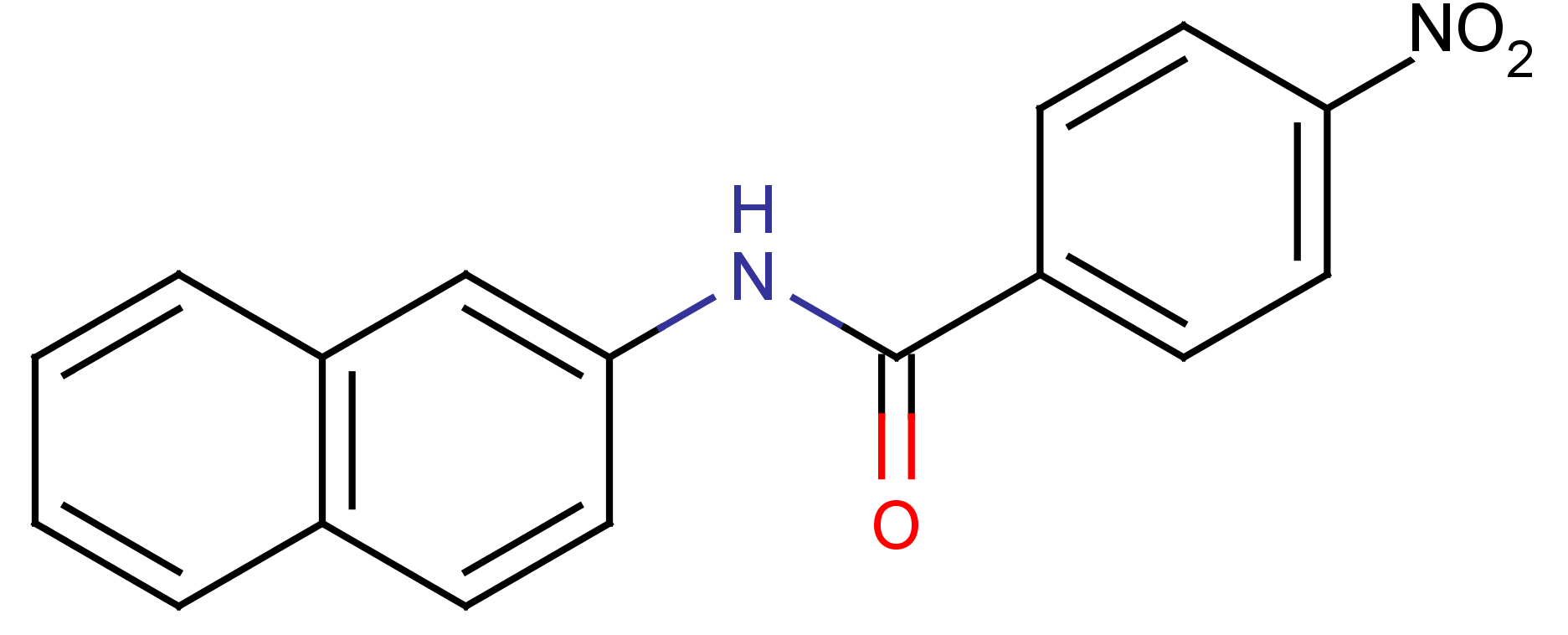** | **mir122** | **Active** | **Active** |
| **1c [1]** | **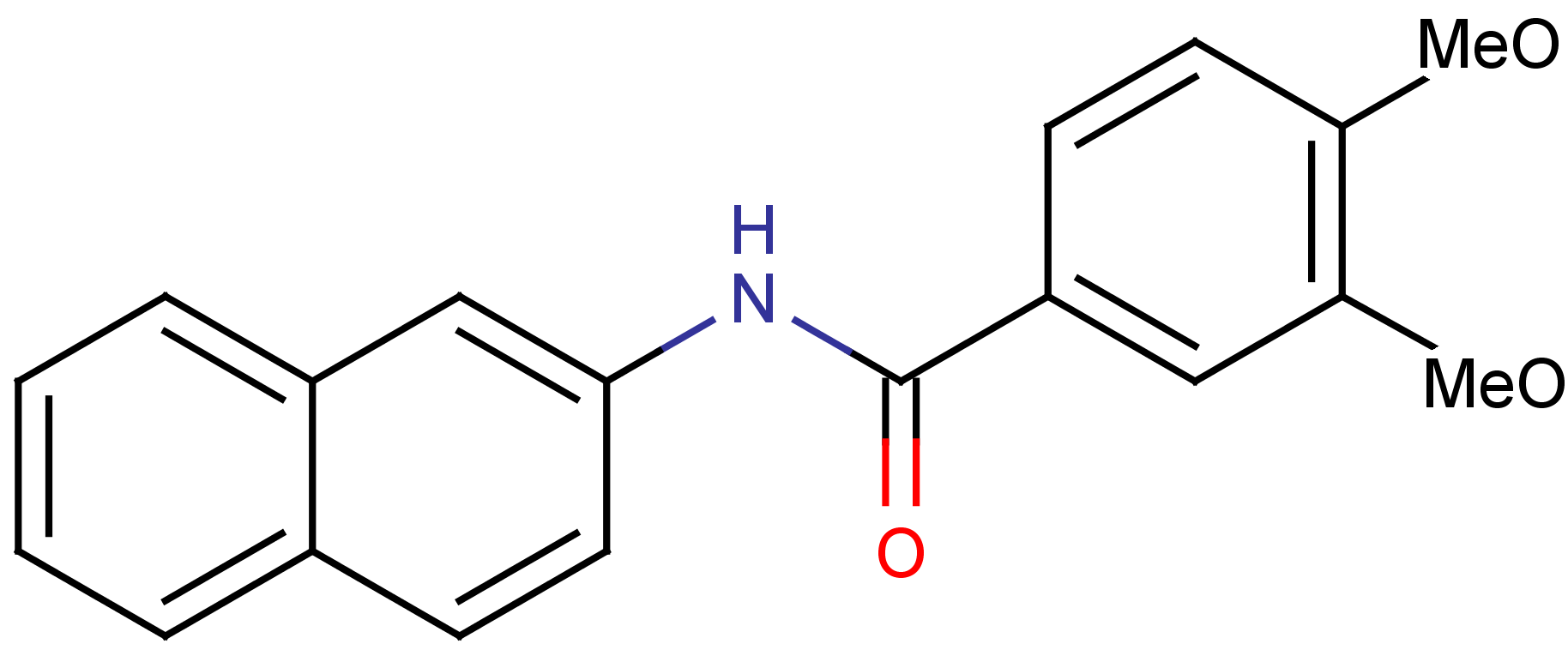** | **mir122** | **Active** | **Active** |
| **1d [1]** | **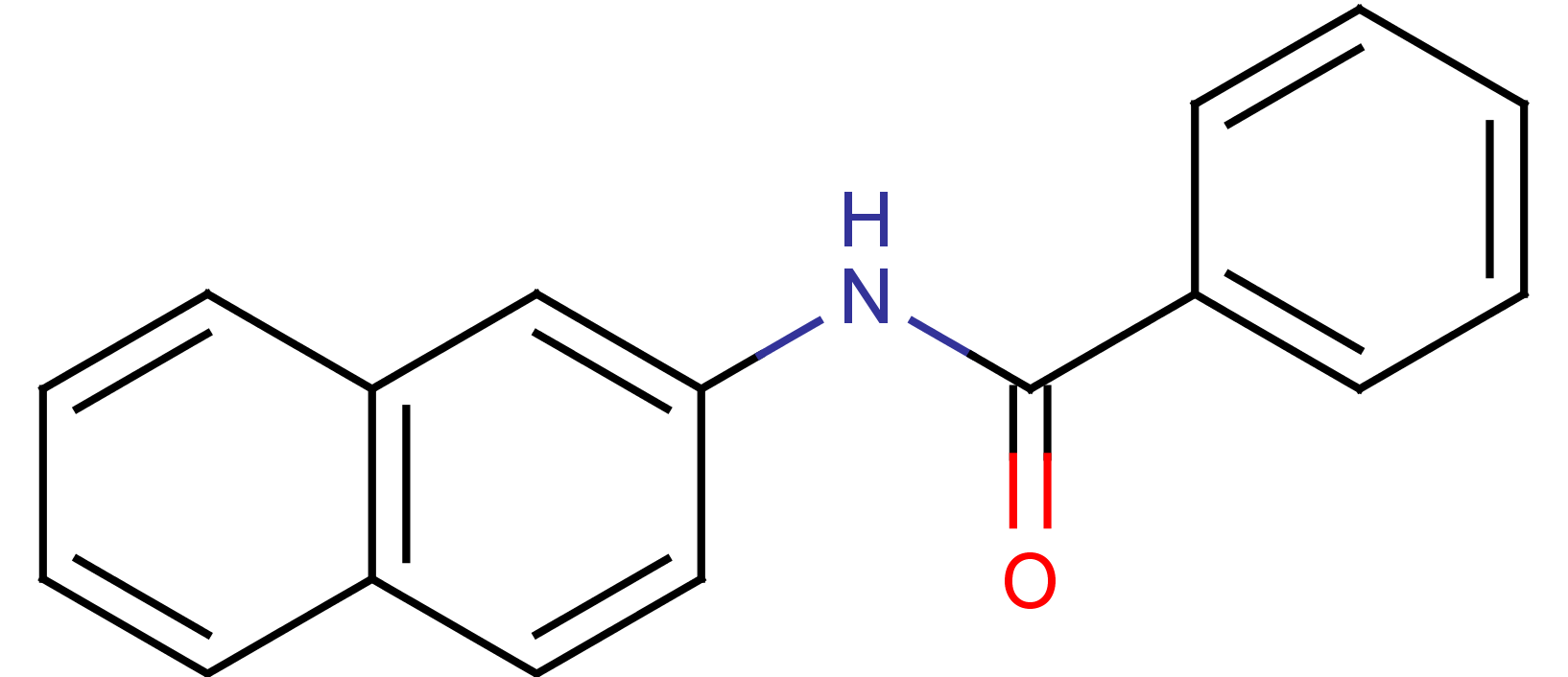** | **mir122** | **Active** | **Active** |
| **1e [1]** | **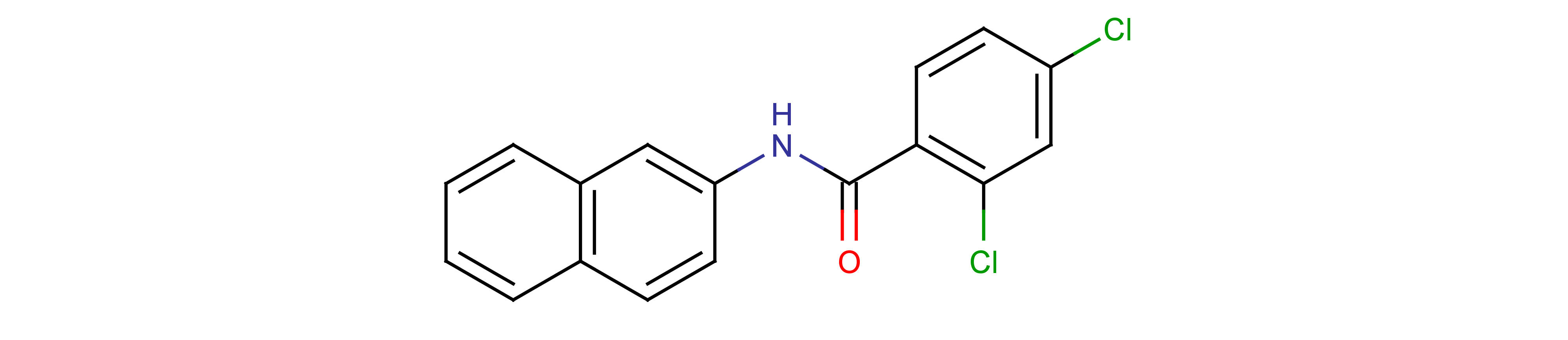** | **mir122** | **Active** | **Active** |
| 1f [1] | 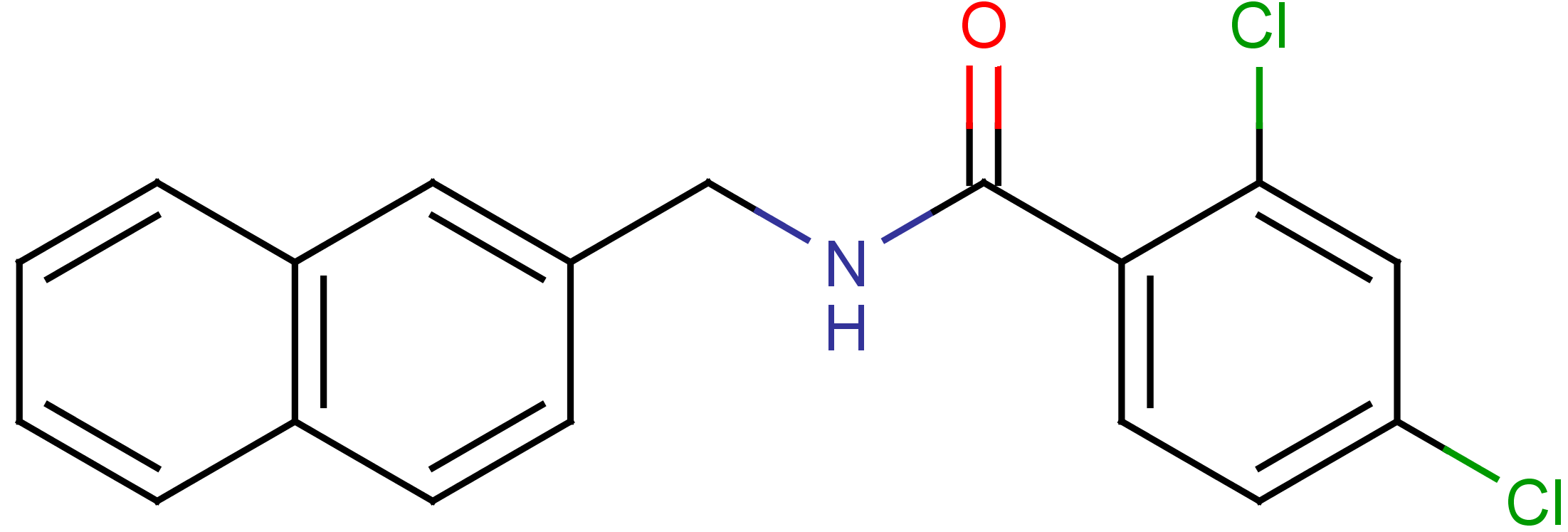 | mir122 | Active | Inactive |
| **1g [1]** | **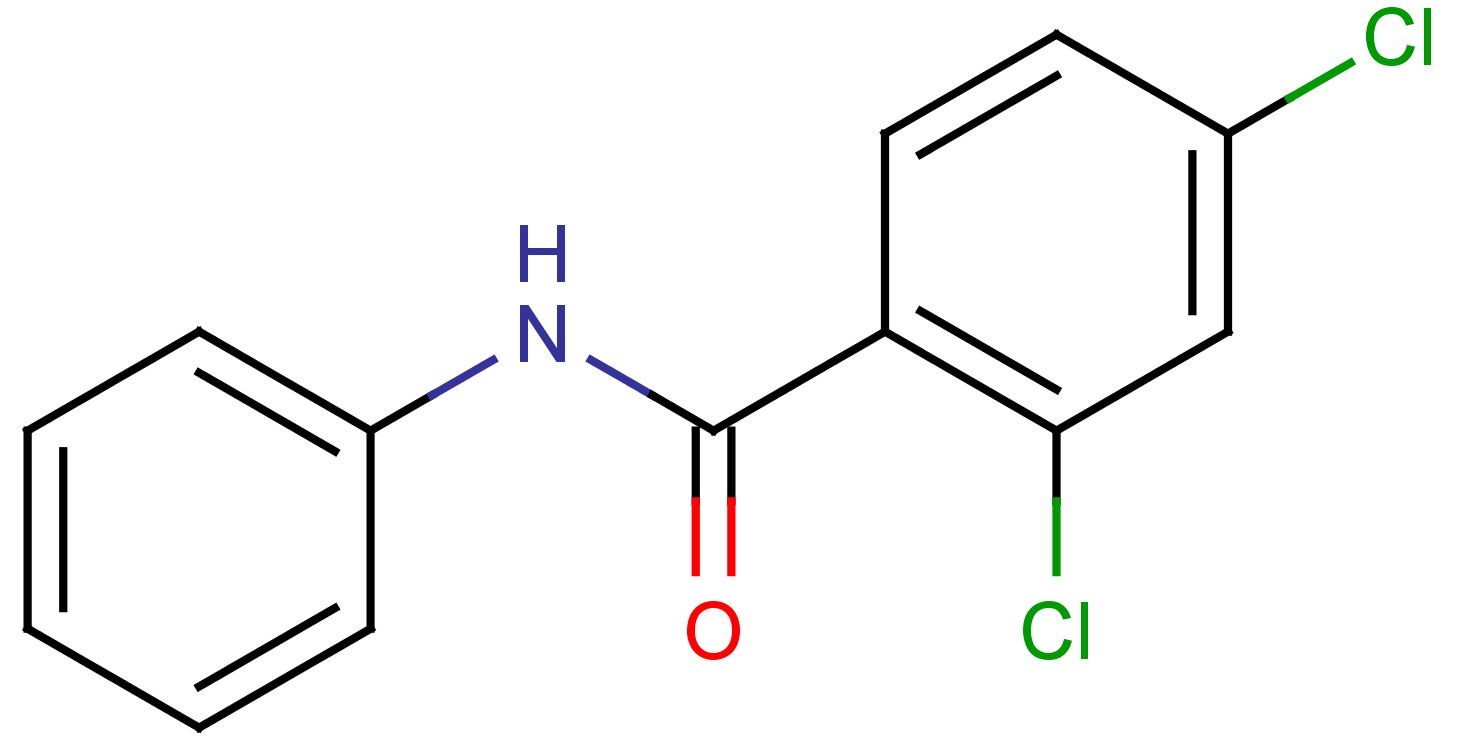** | **mir122** | **Active** | **Active** |
| **1h [1]** | **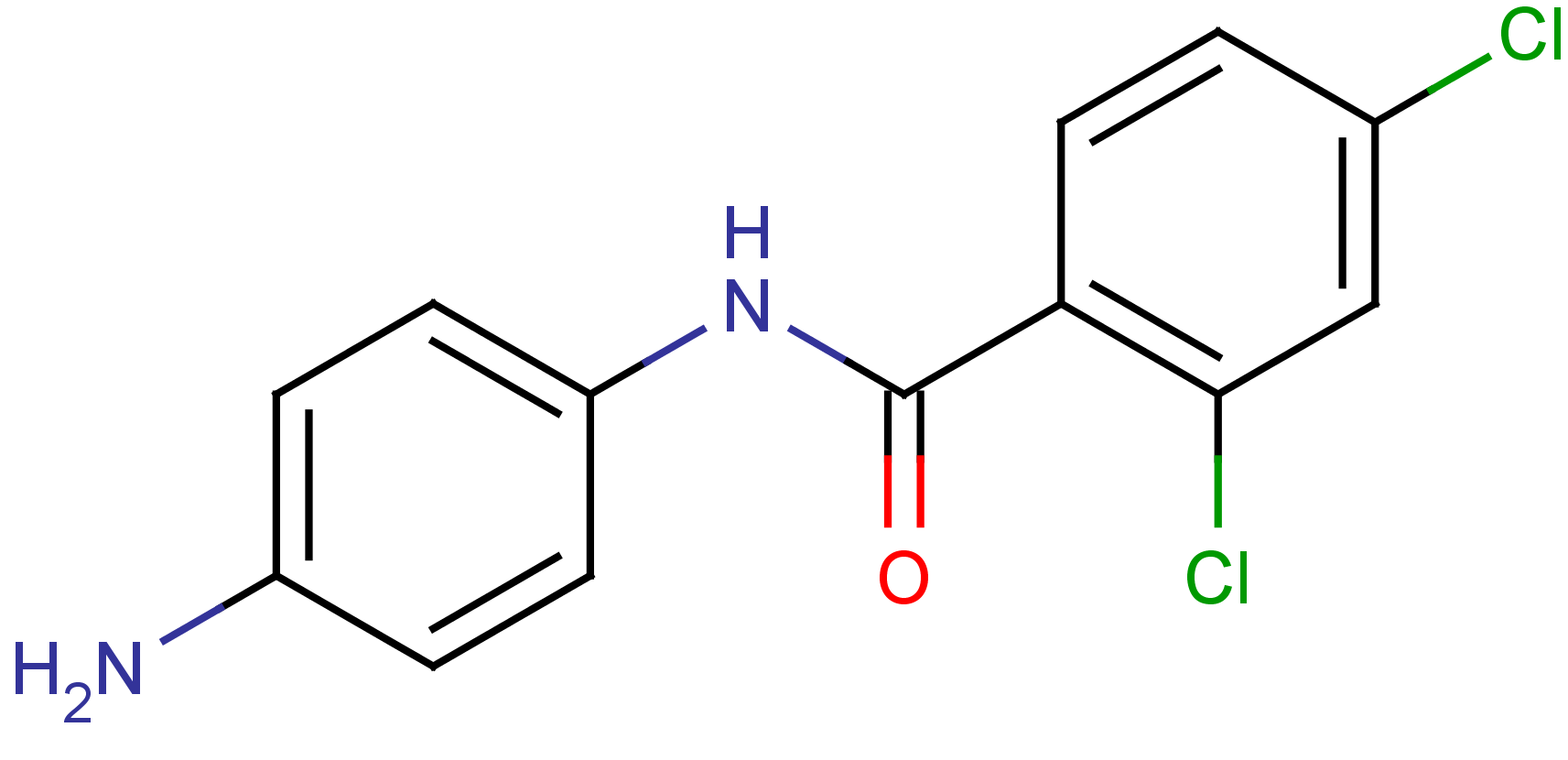** | **mir122** | **Active** | **Active** |
| **1i [1]** | **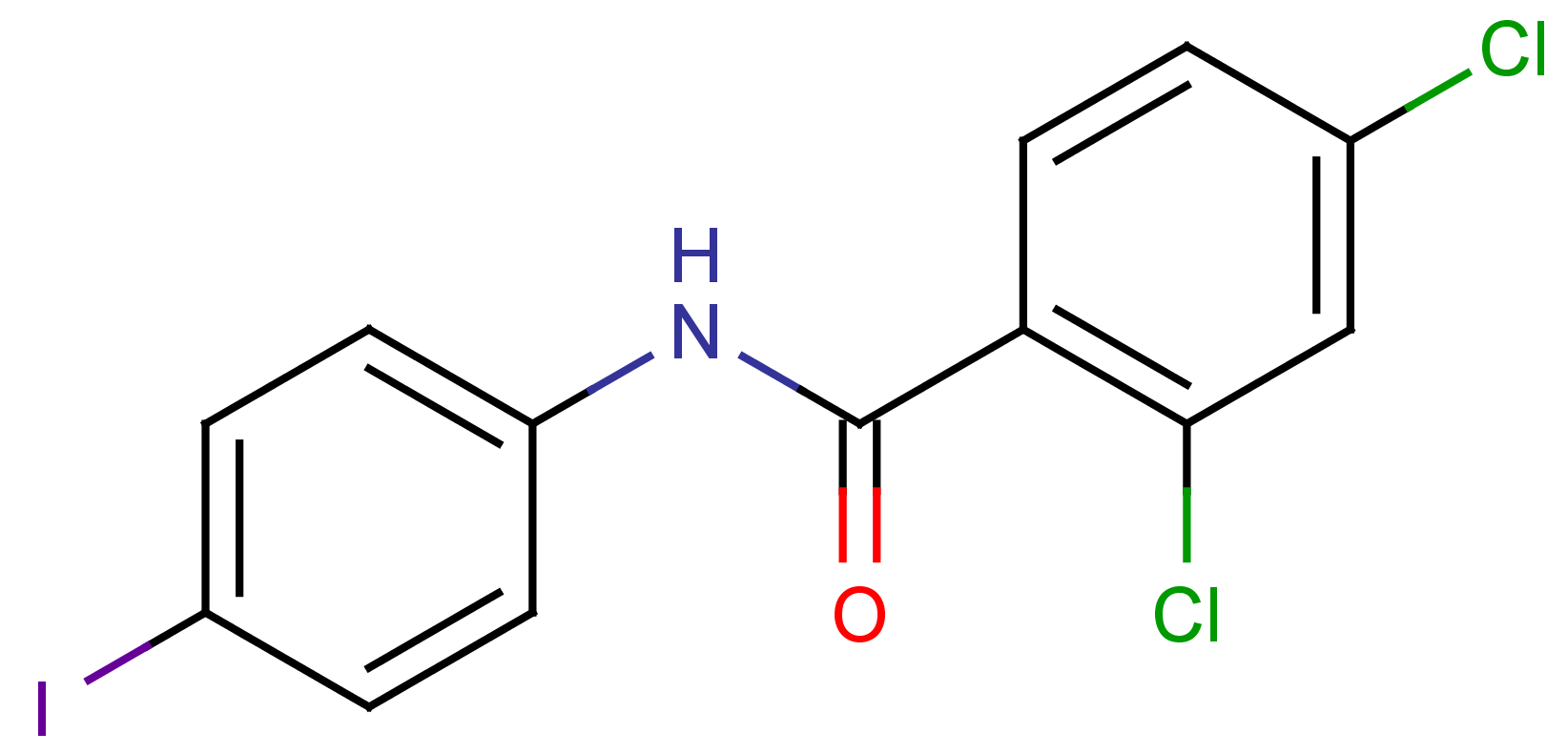** | **mir122** | **Active** | **Active** |
| **1j [1]** | **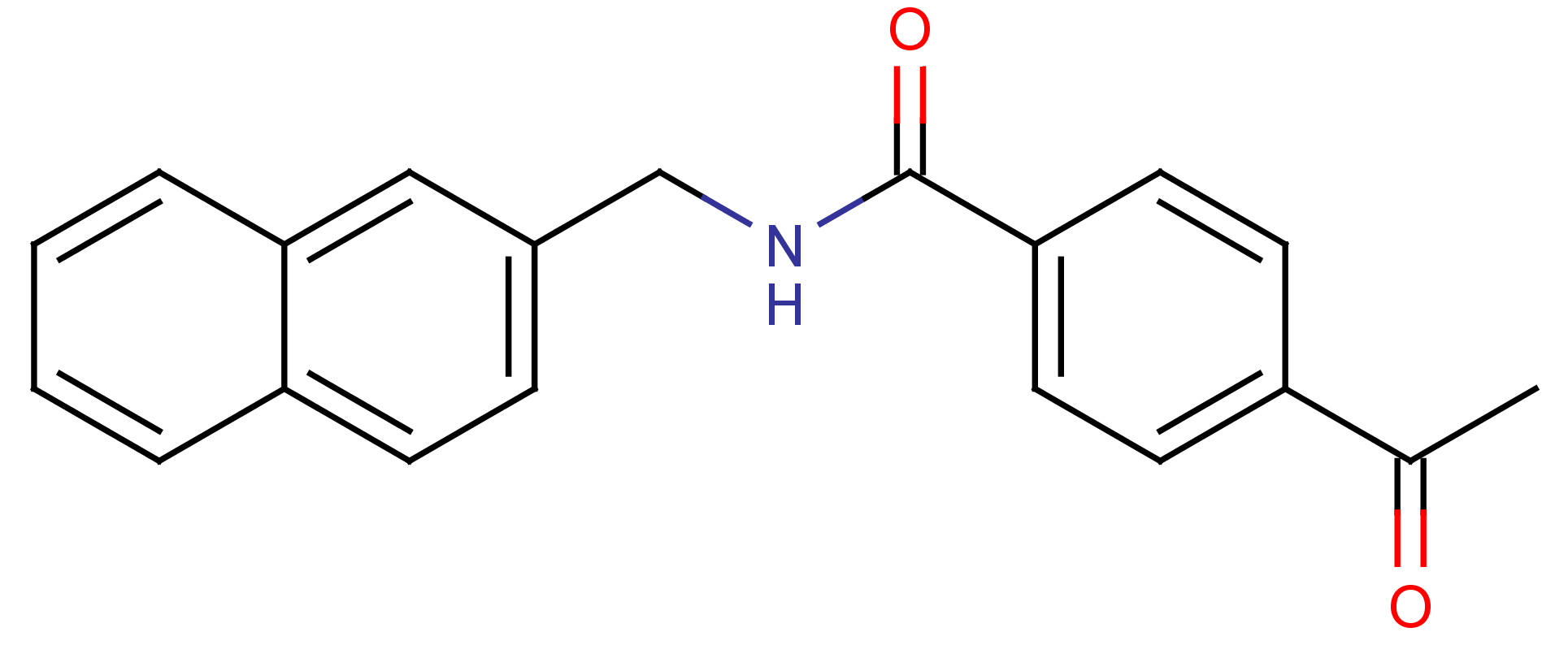** | **mir122** | **Active** | **Active** |
| **1k [1]** | **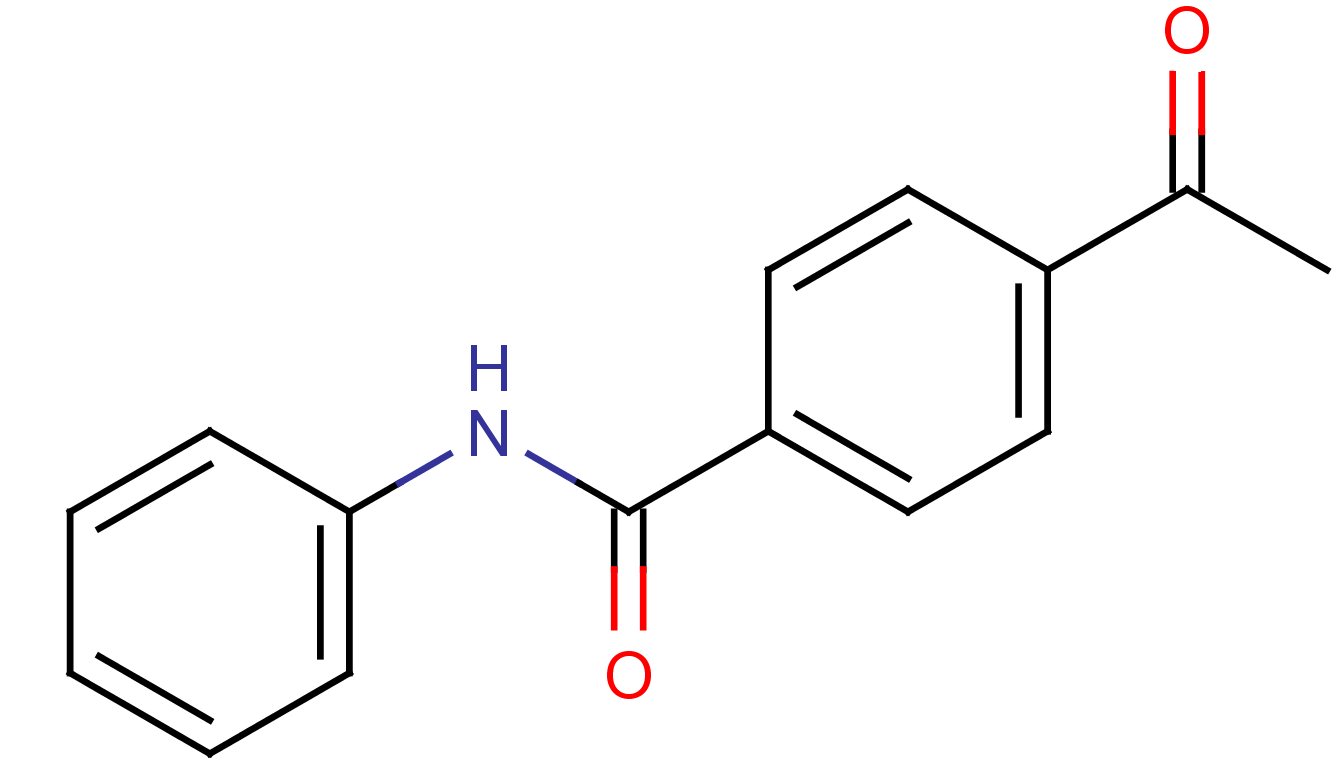** | **mir122** | **Active** | **Active** |
| 1l [1] | 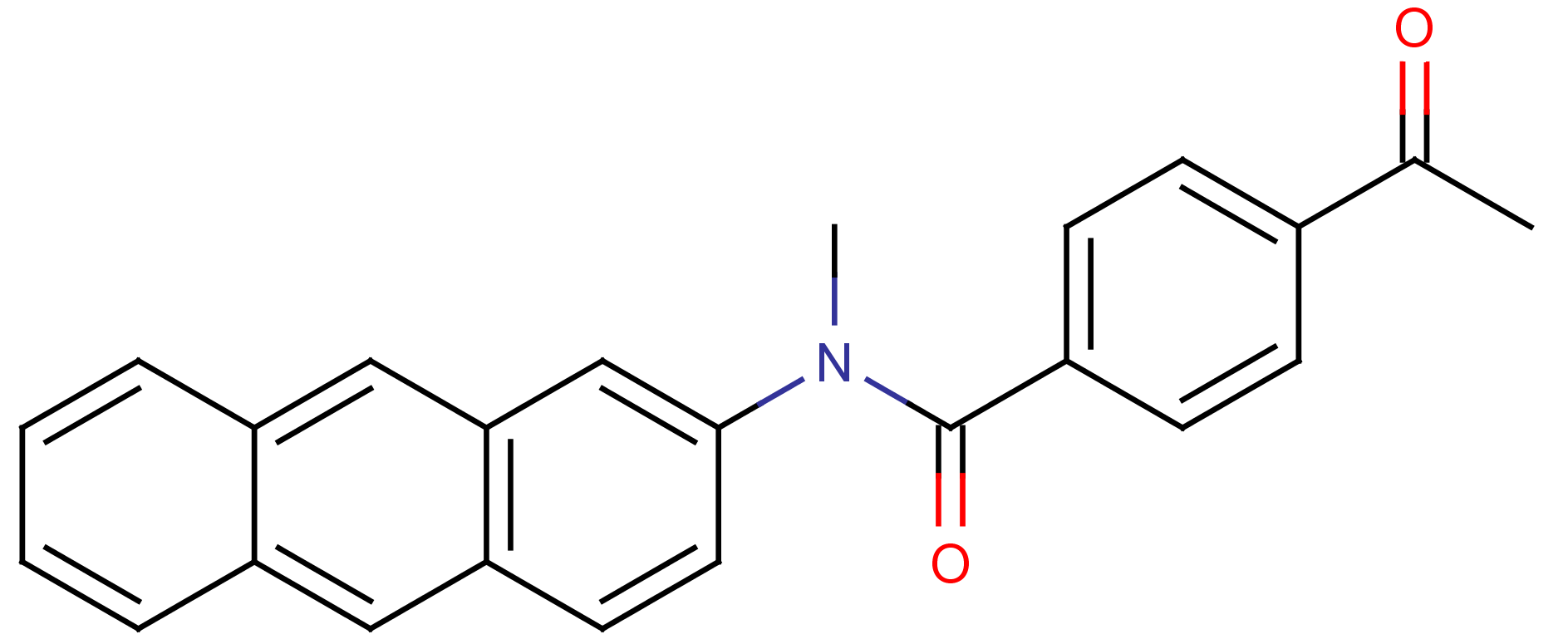 | mir122 | Inactive | Inactive |
| **1m [1]** | **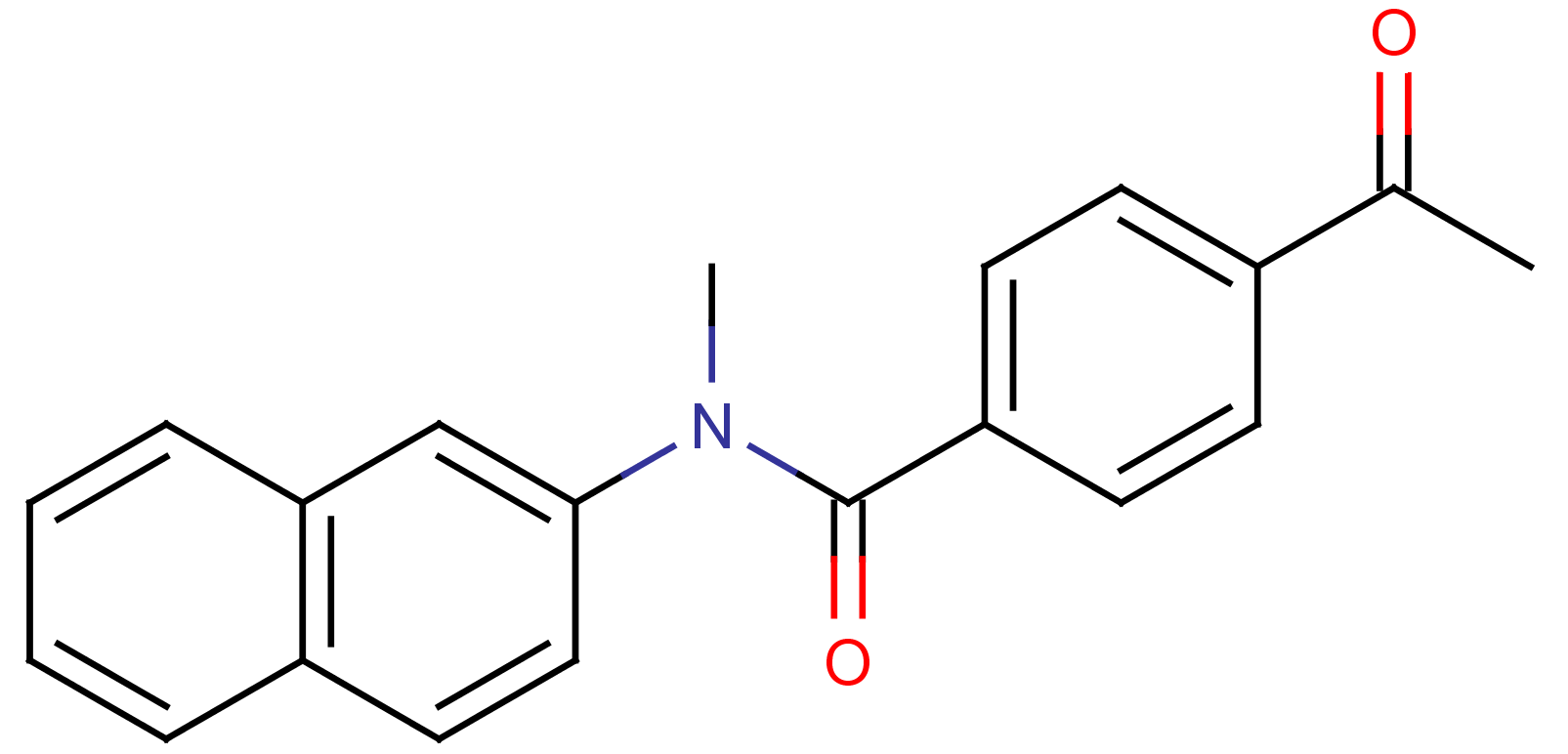** | **mir122** | **Active** | **Active** |
| 2a [1] | 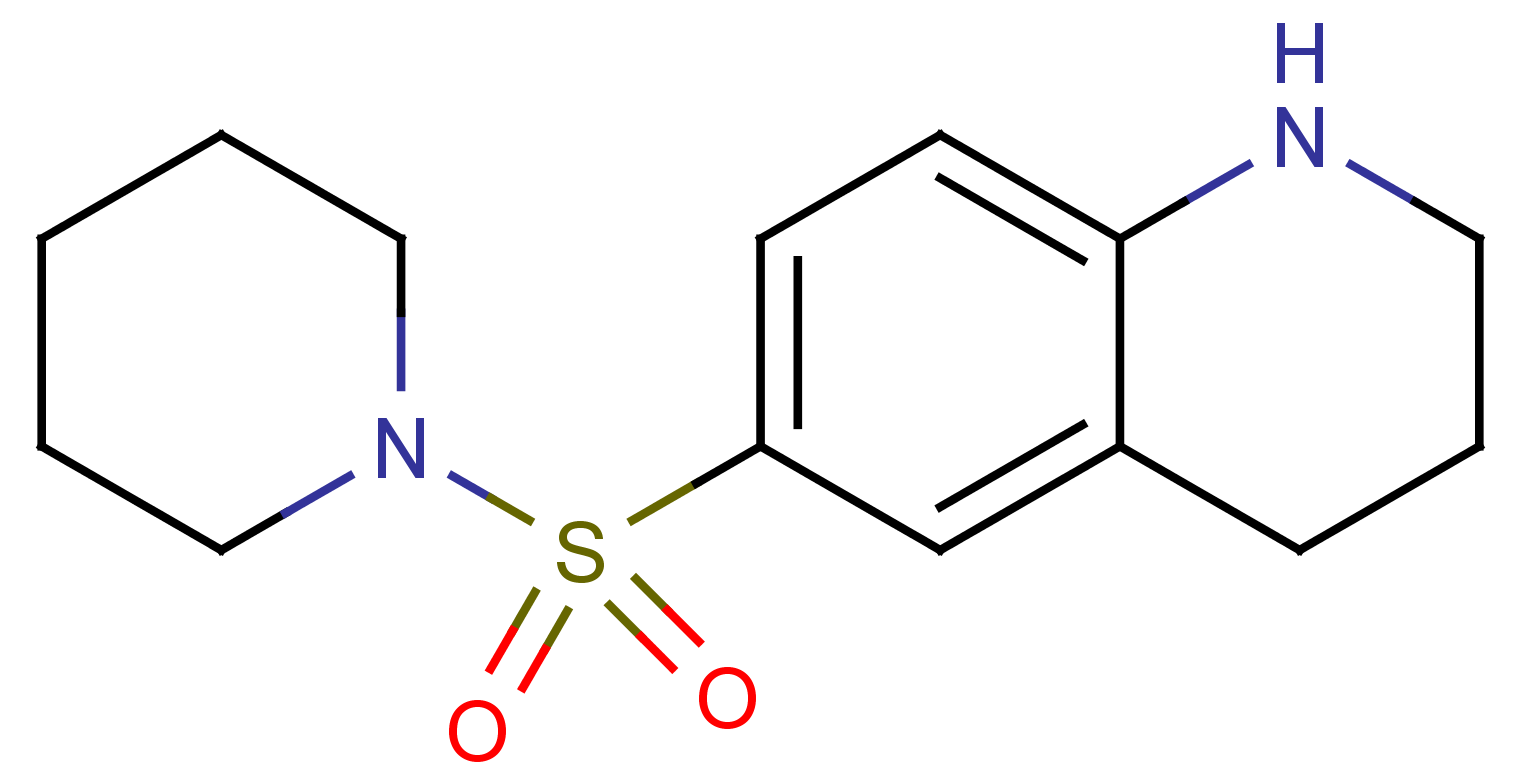 | mir122 | Inactive | Inactive |
| 2b [1] | 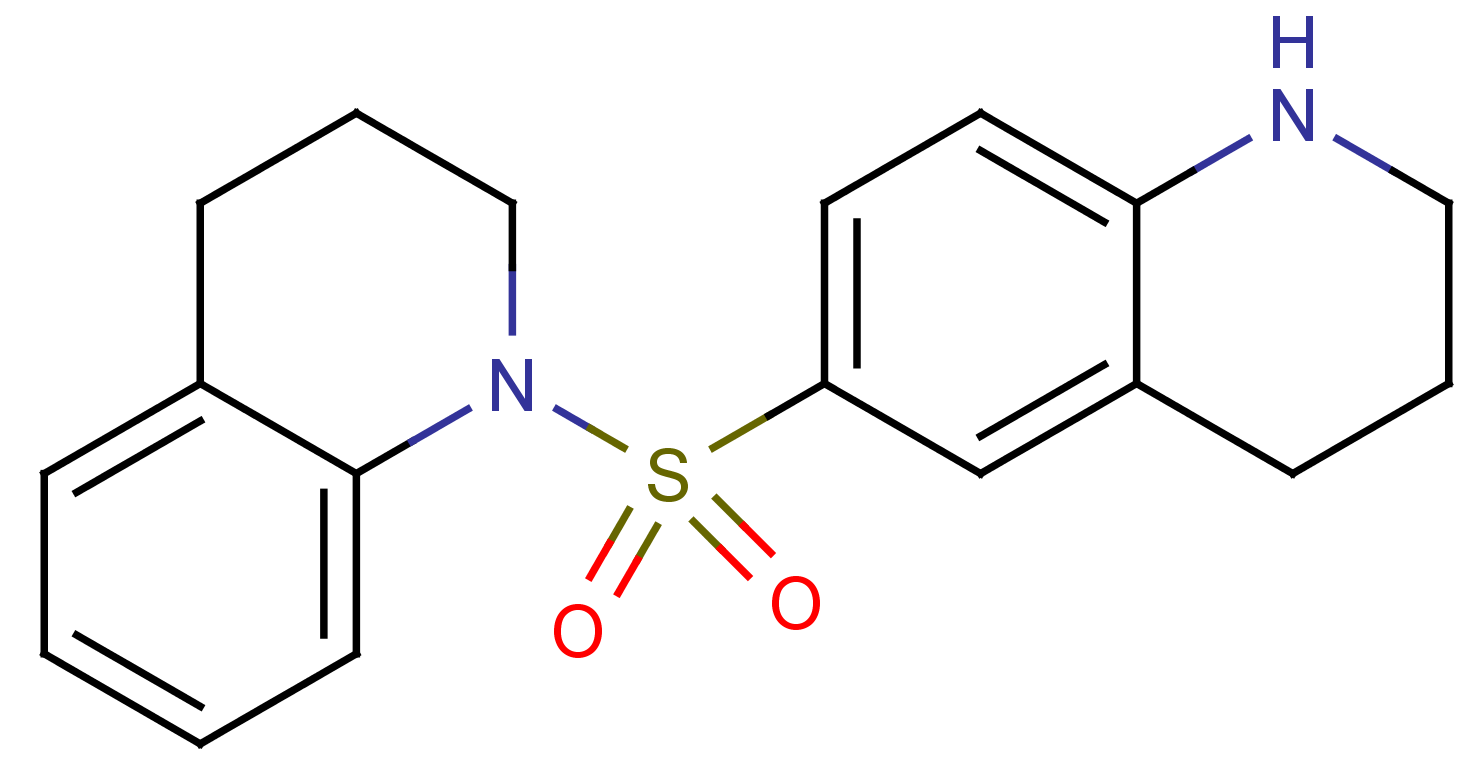 |  | Inactive | Inactive |
| 2c [1] | 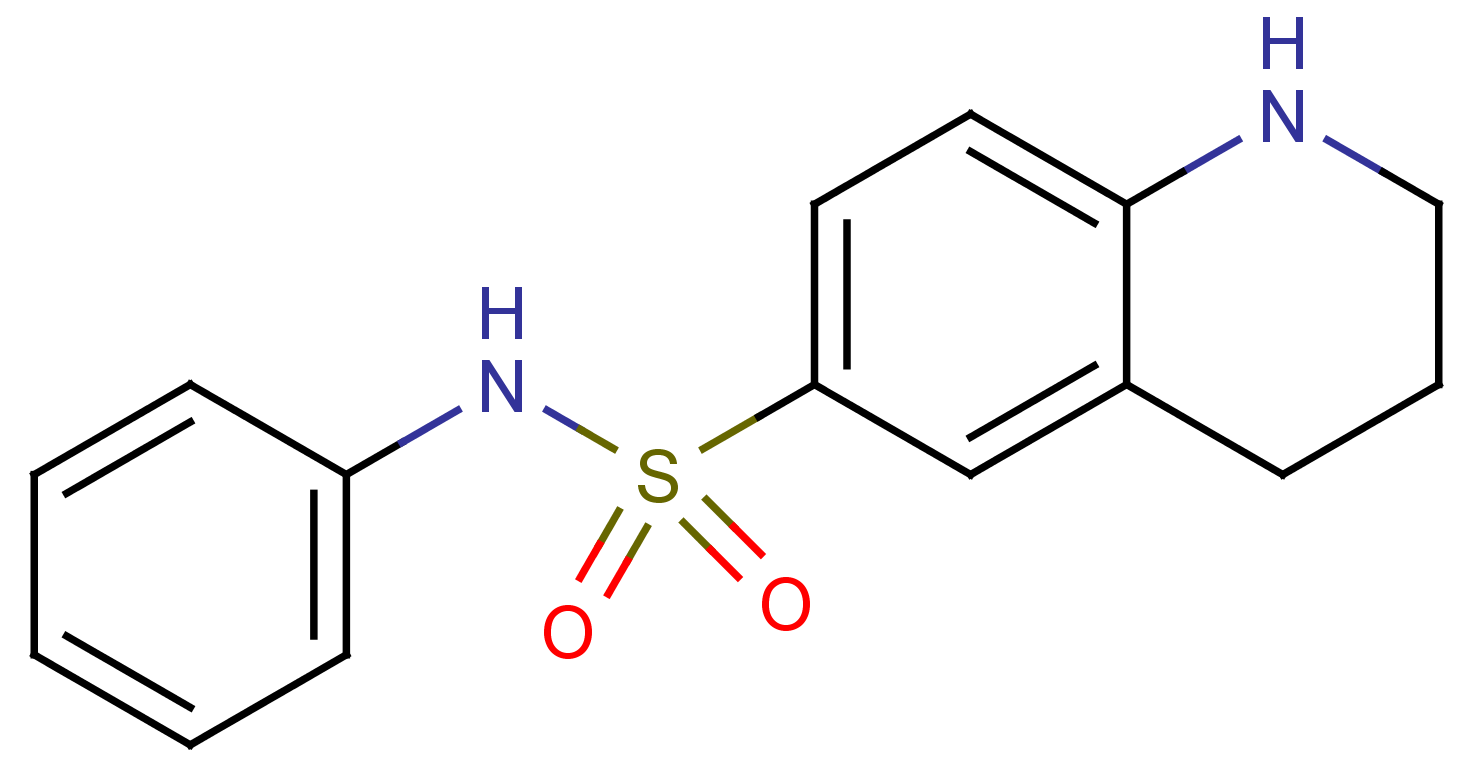 | mir122 | Inactive | Inactive |
| 2d [1] | 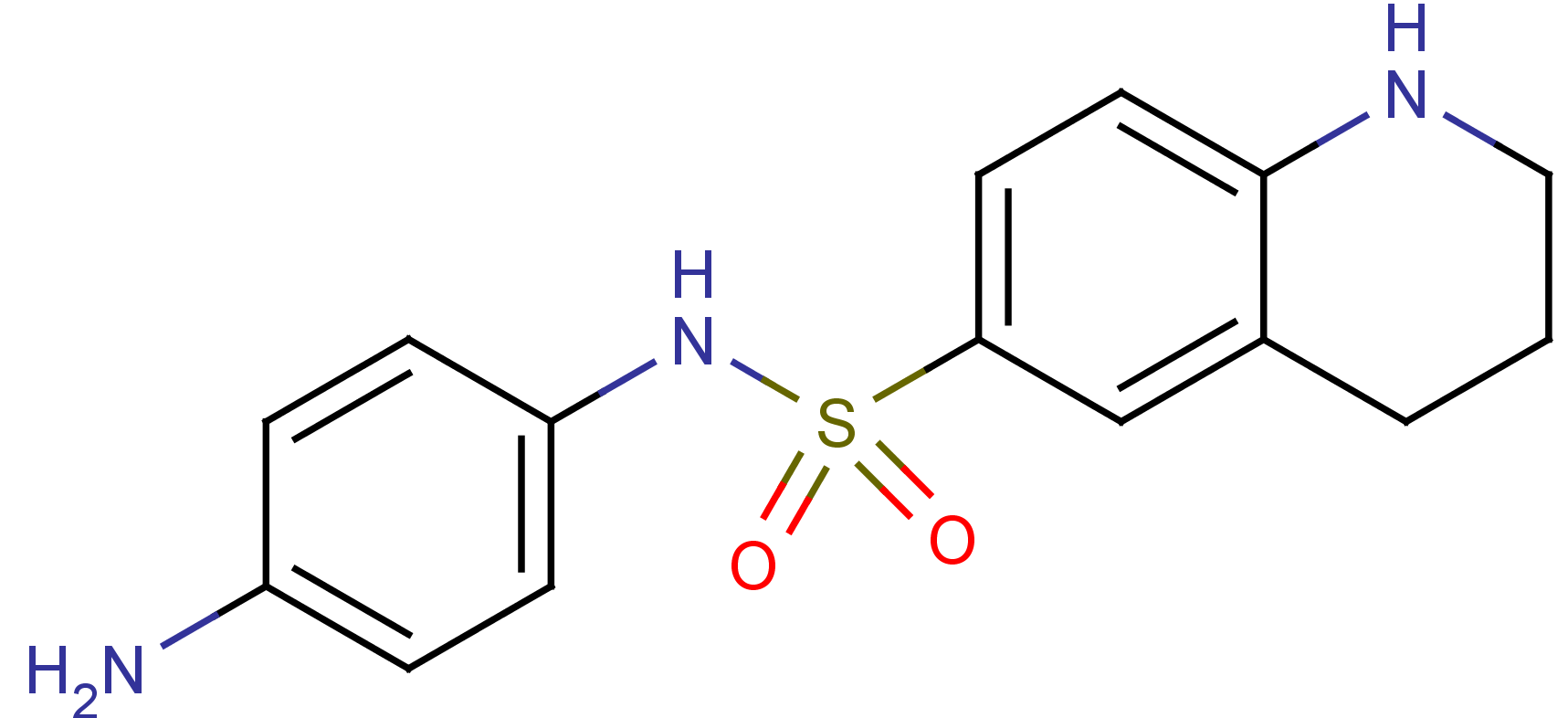 | mir122 | Inactive | Inactive |
| 2e [1] | 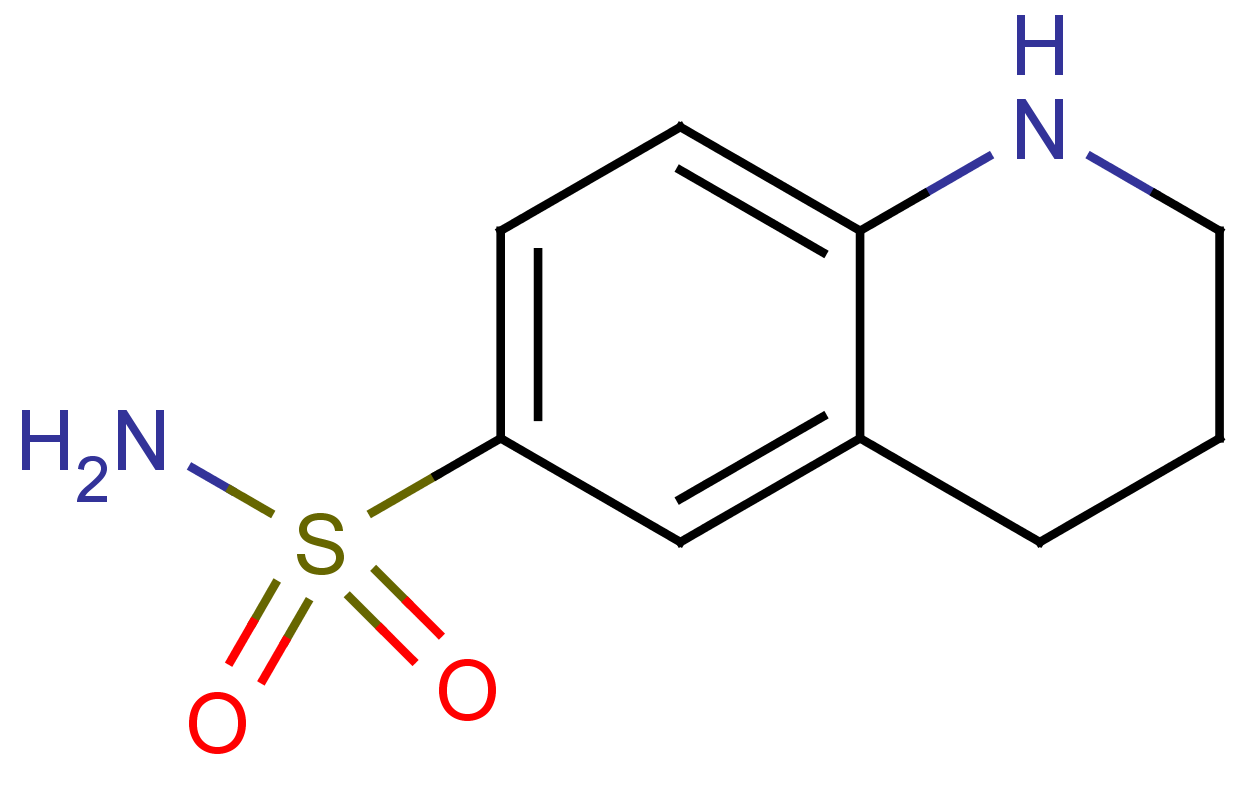 | mir122 | Inactive | Inactive |
| 2f [1] | 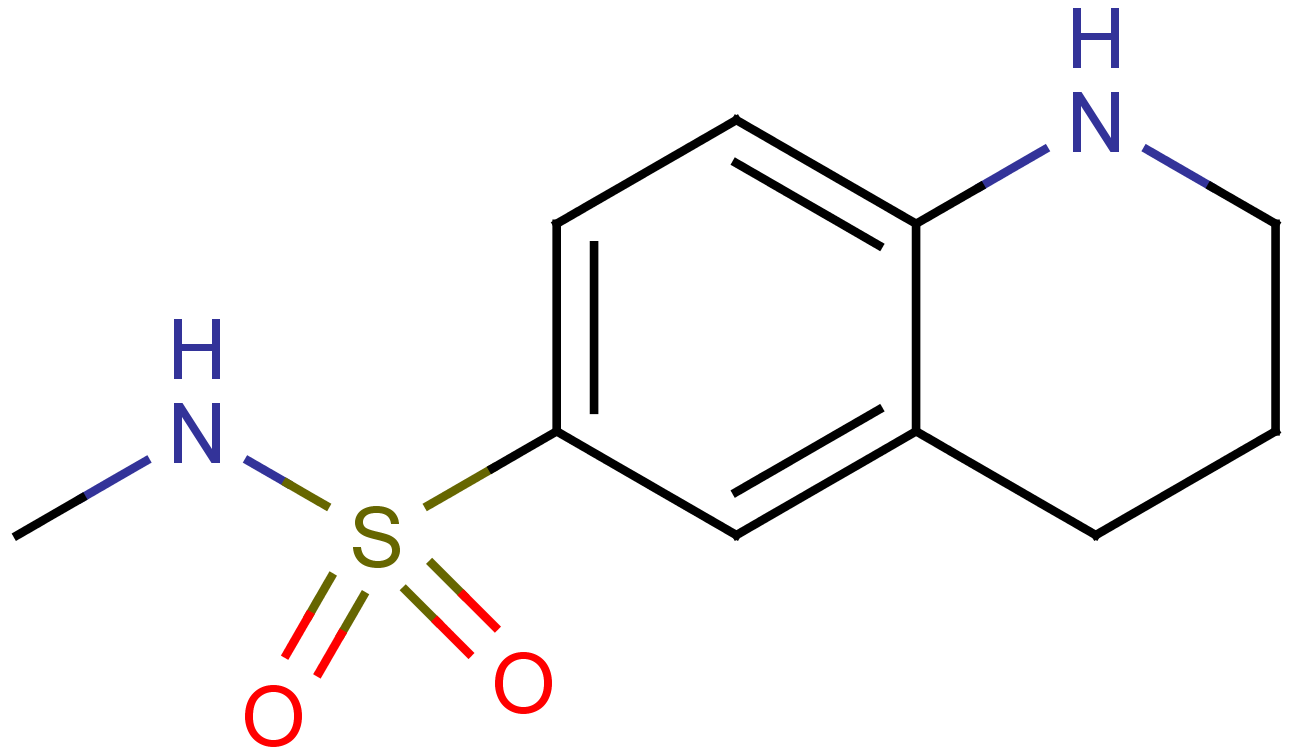 | mir122 | Inactive | Inactive |
| 2g [1] | 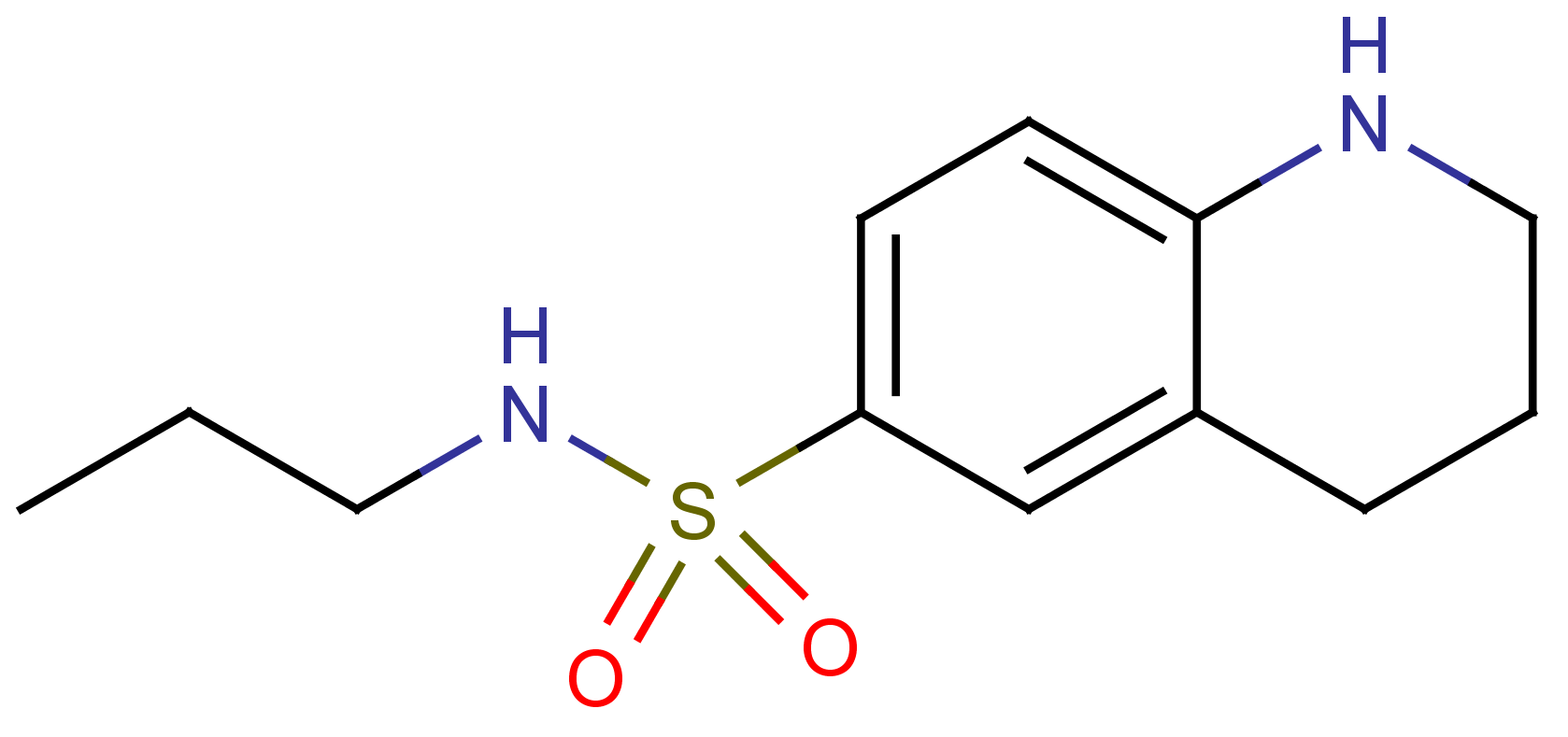 | mir122 | Inactive | Inactive |
| 2h [1] | 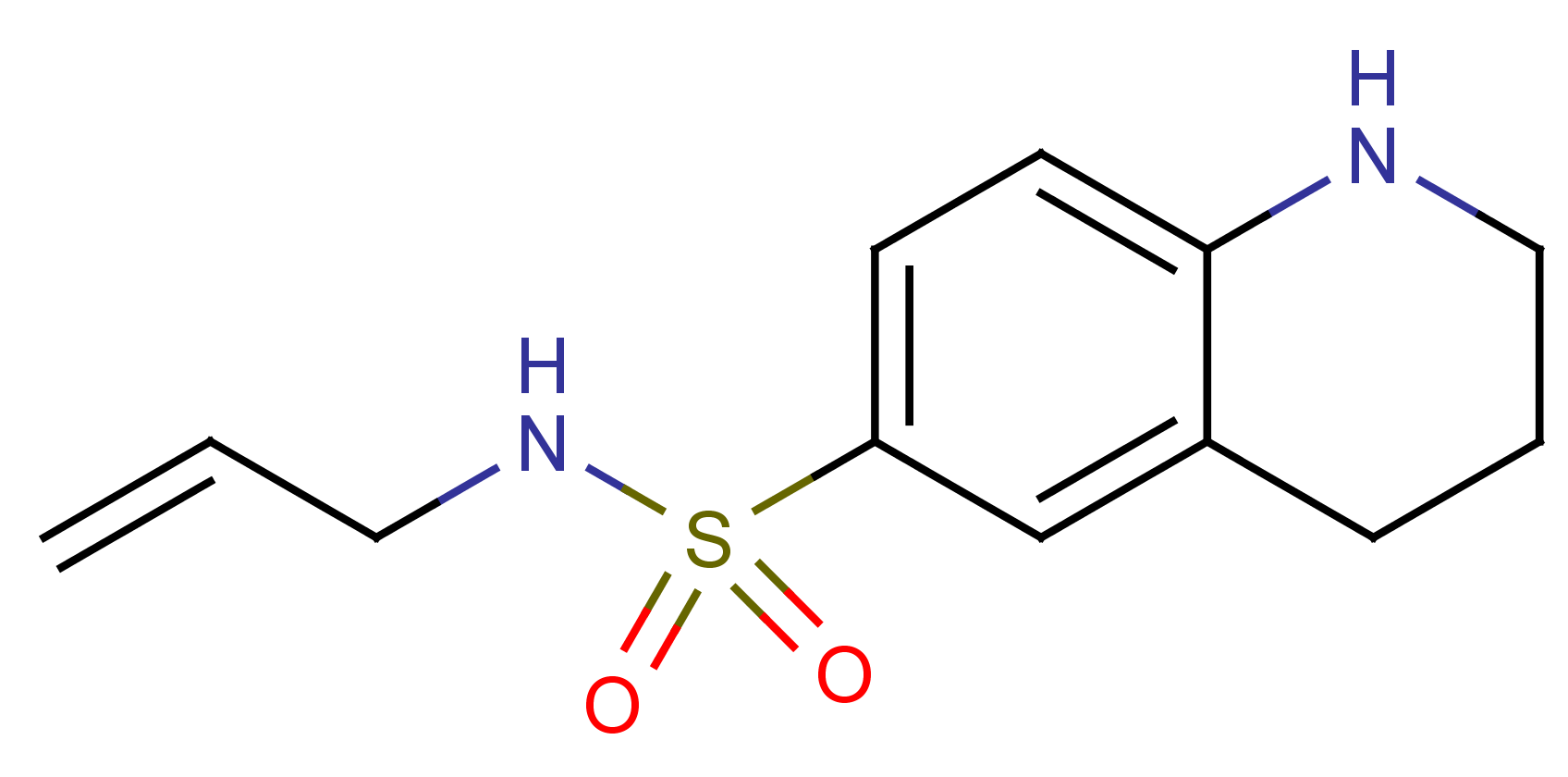 | mir122 | Inactive | Inactive |
| 2i [1] | 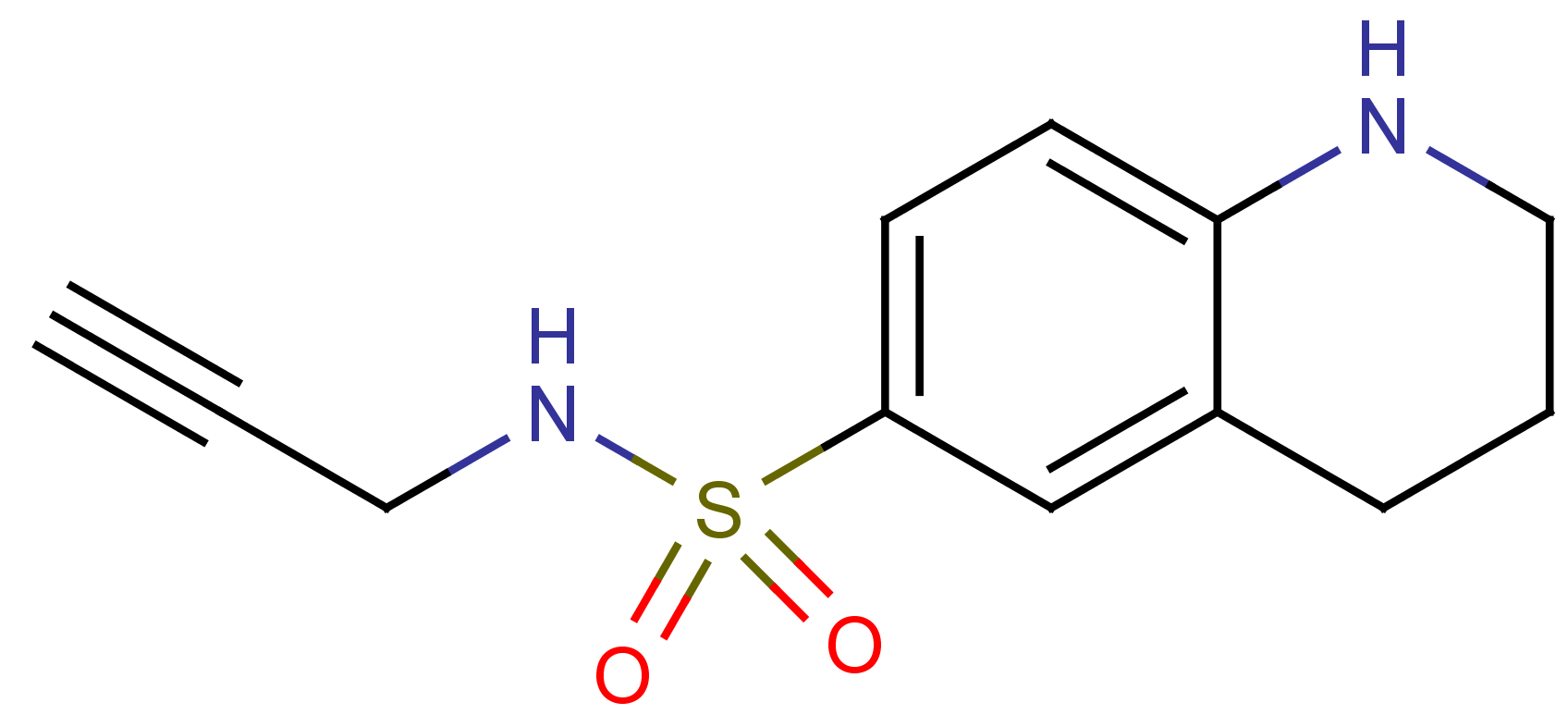 | mir122 | Inactive | Inactive |
| 2j [1] | 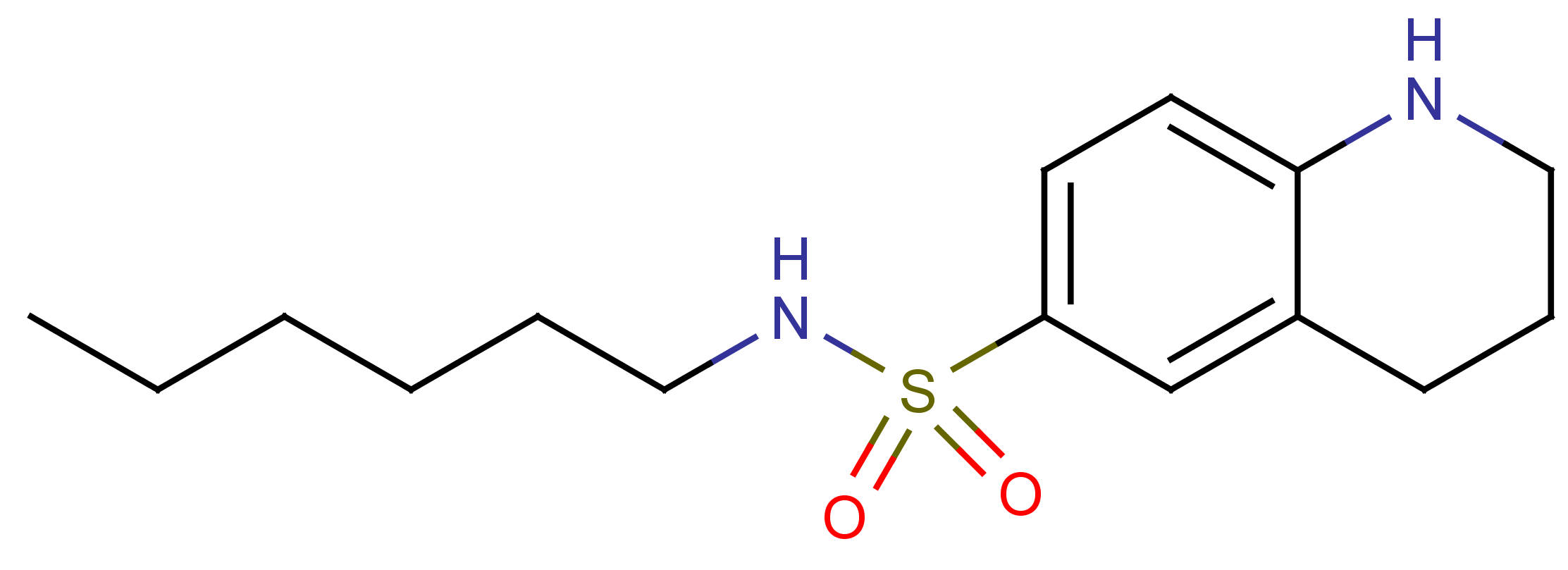 | mir122 | Inactive | Inactive |
| 3a [1] | 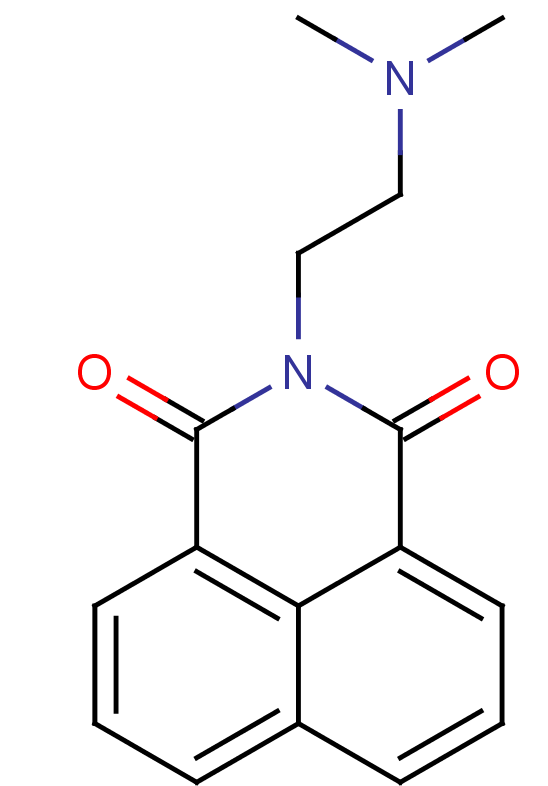 | mir122 | Inactive | Inactive |
| 3b [1] | 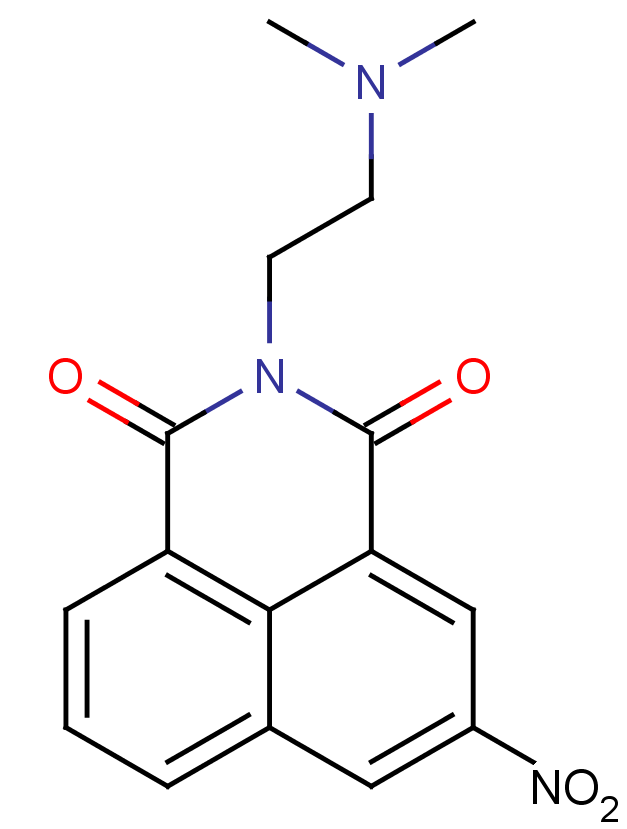 | mir122 | Inactive | Inactive |
| 3c [1] | 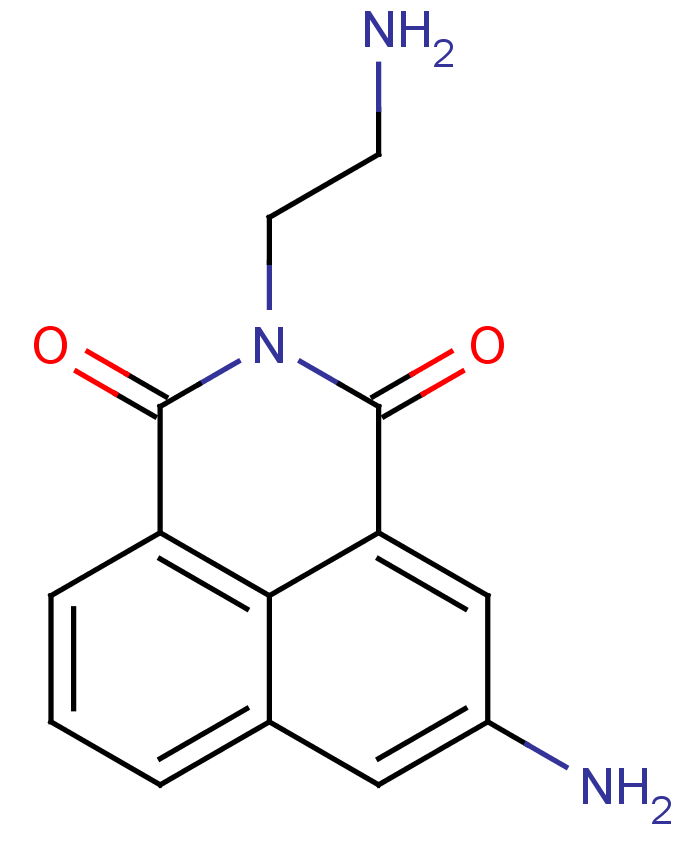 | mir122 | Inactive | Inactive |
| 3d [1] | 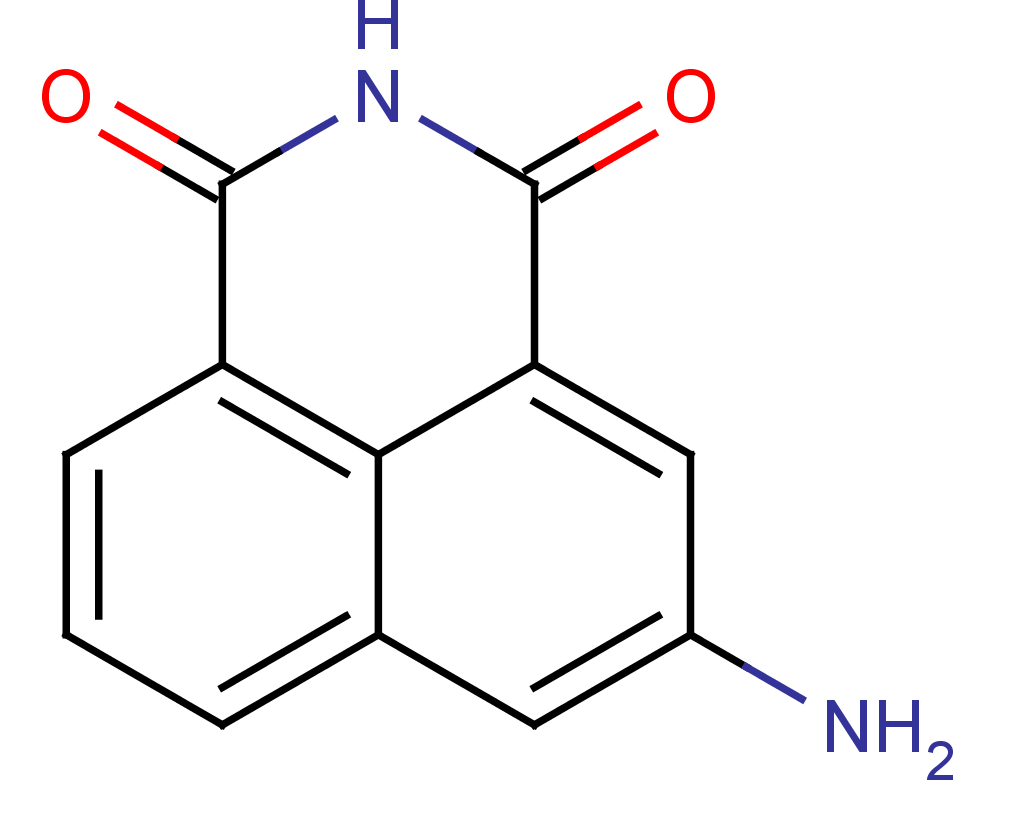 | mir122 | Inactive | Inactive |
| 3e [1] | 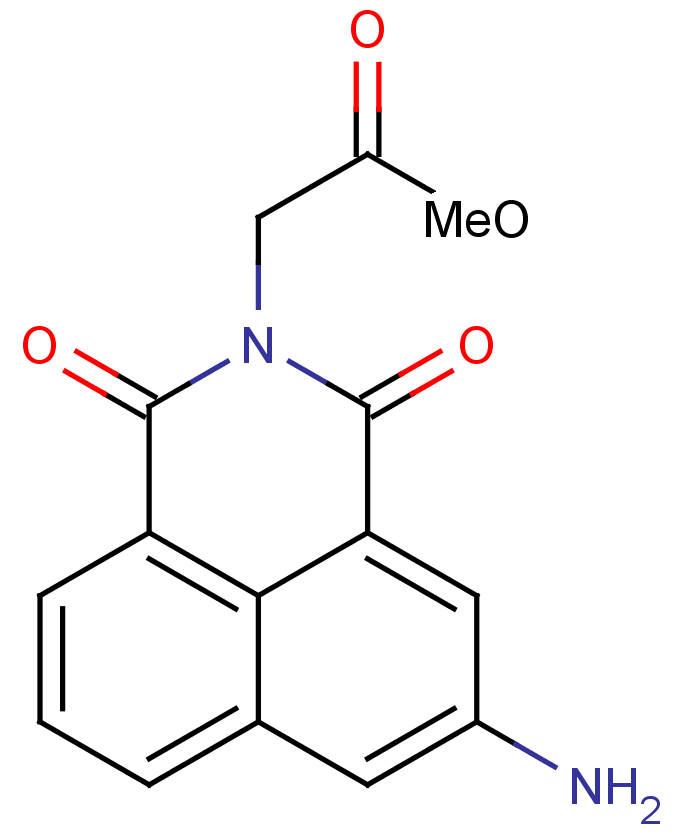 | mir122 | Inactive | Inactive |
| 3f [1] | 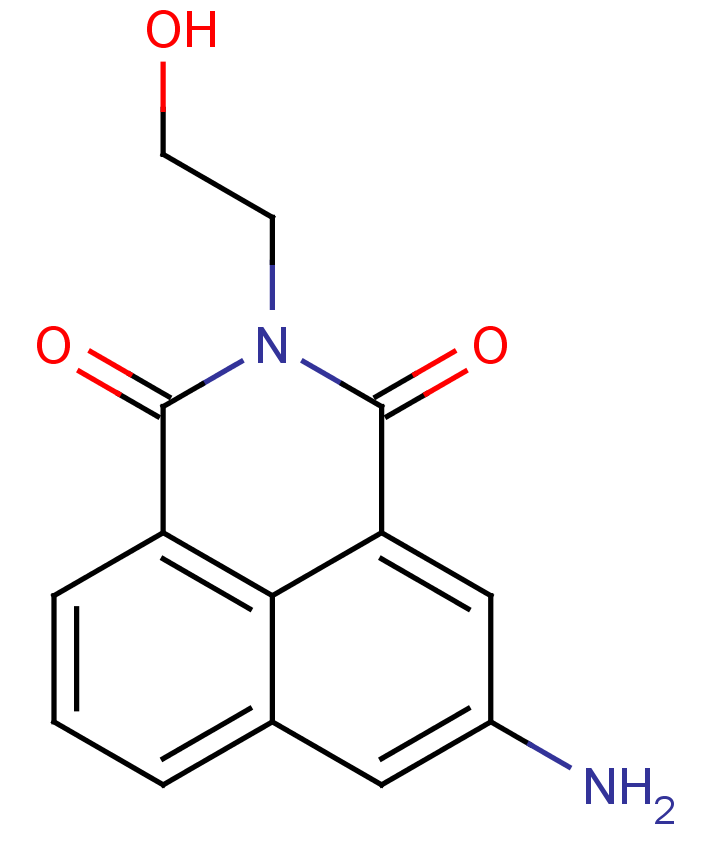 | mir122 | Inactive | Inactive |
| 3g [1] | 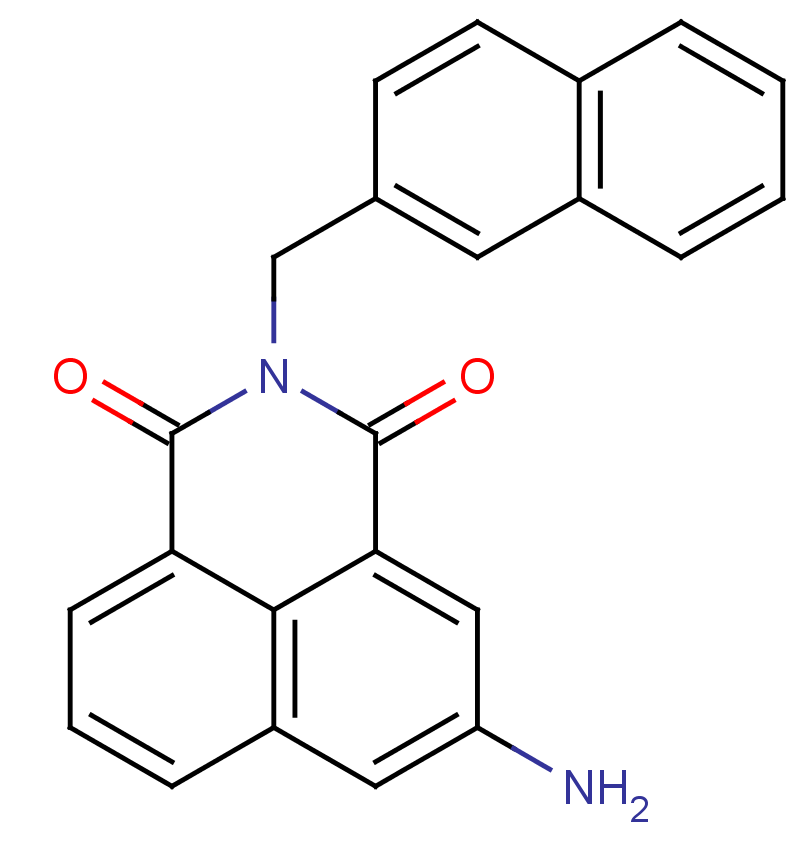 | mir122 | Inactive | Inactive |
| CID2259 [2] | 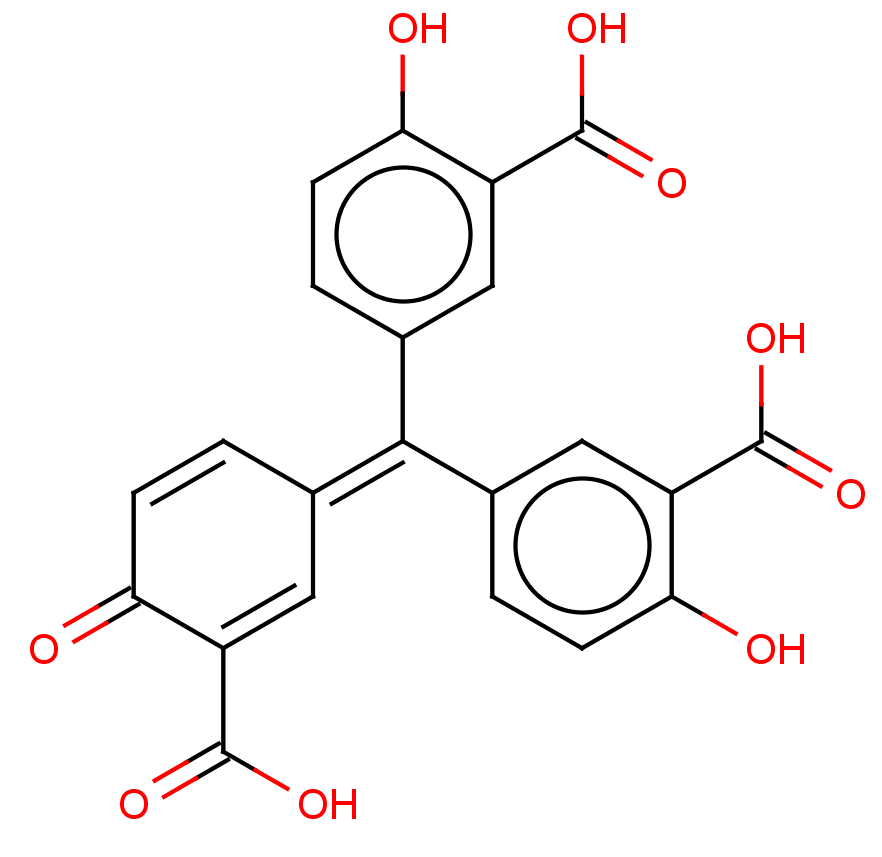 | siRNA | Inactive | Inactive |
| CID3229 [3] | 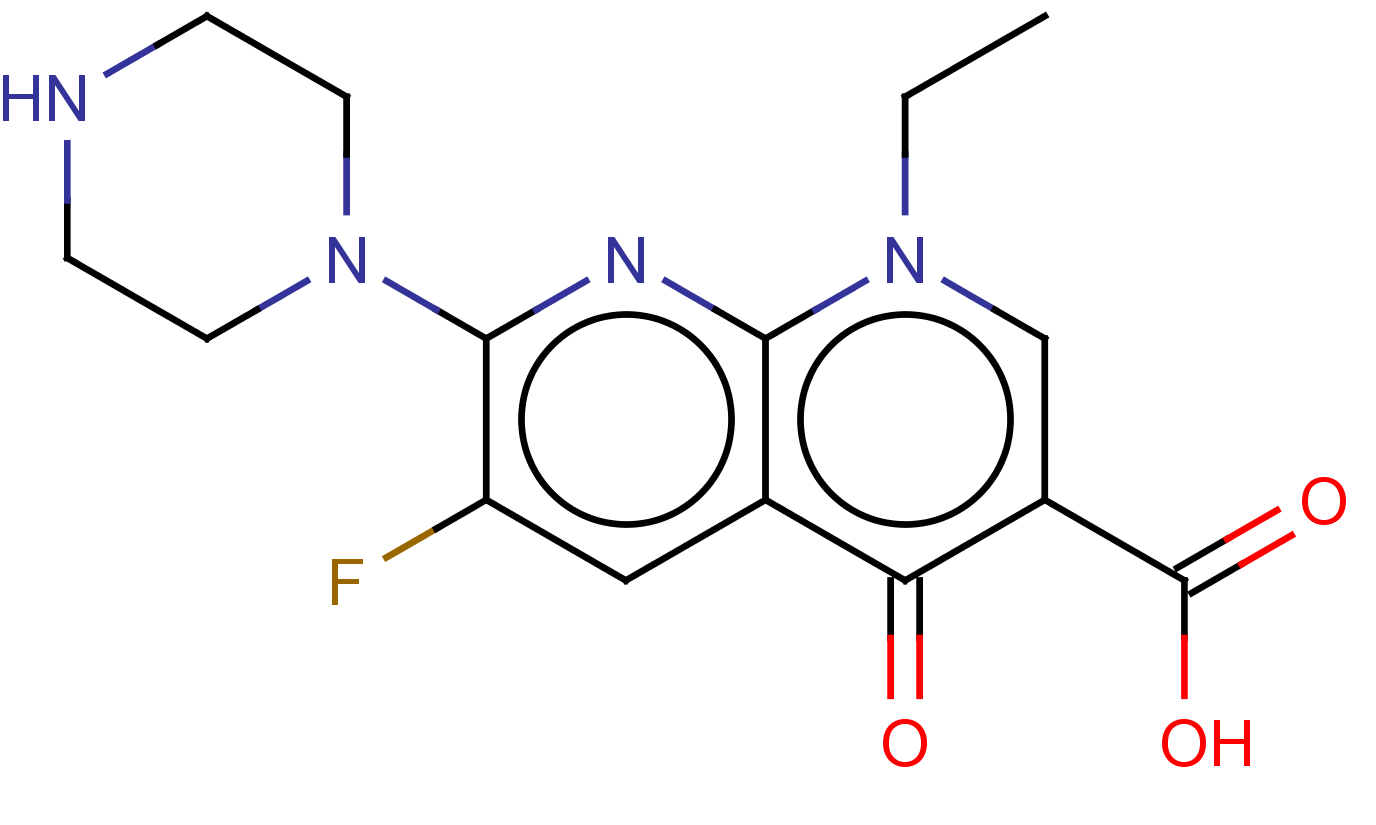 | miRNA with tumour suppressor function | Inactive | Inactive |
| CID4624 [2] | 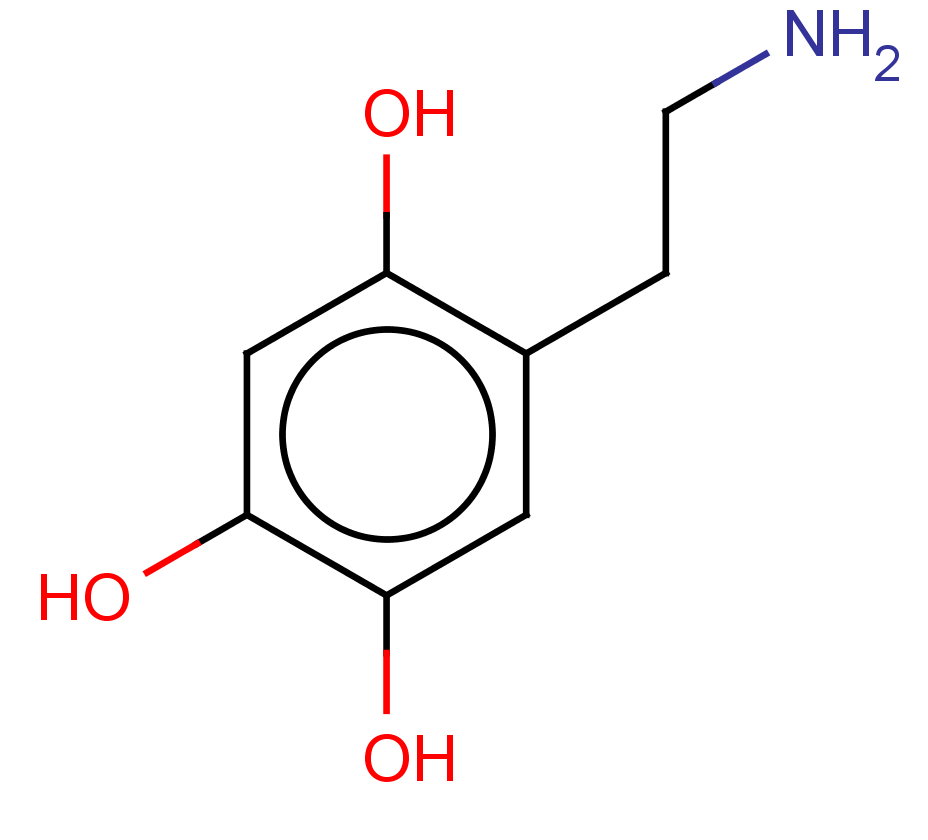 | siRNA | Inactive | Inactive |
| CID8514 [2] | 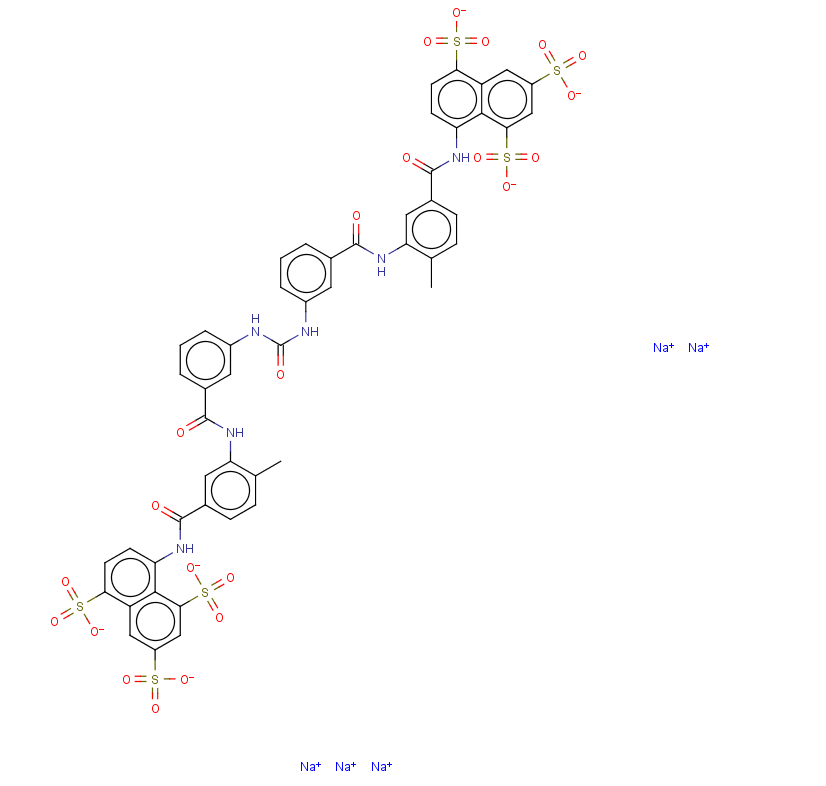 | siRNA | Inactive | Inactive |
| CID19649 [4] | 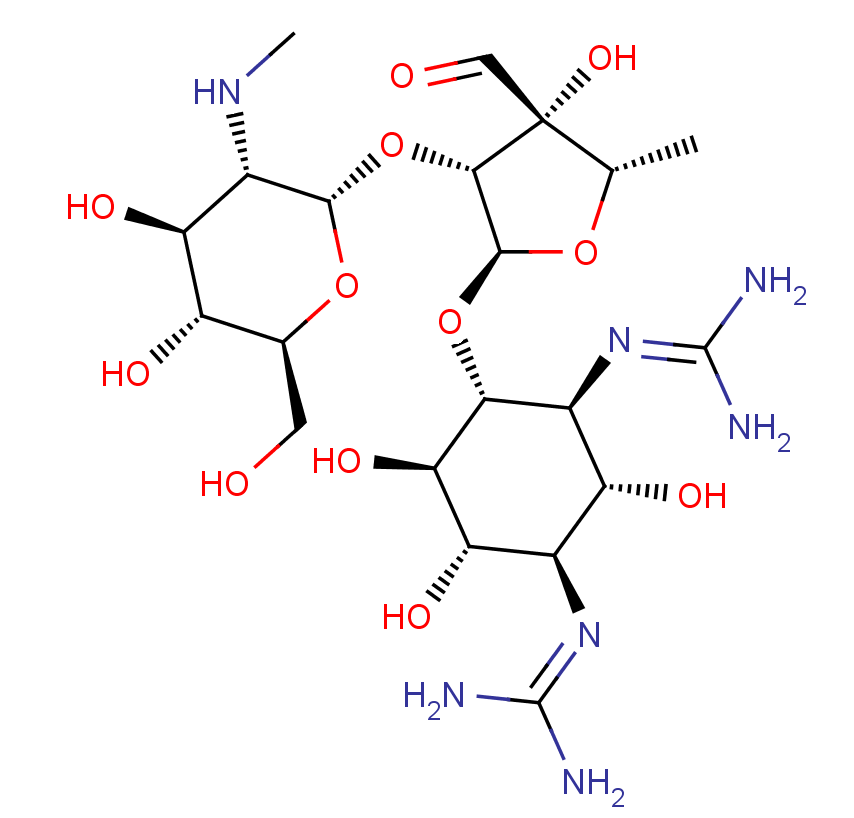 | mir-21 | Inactive | Inactive |
| CID22394 [5] | 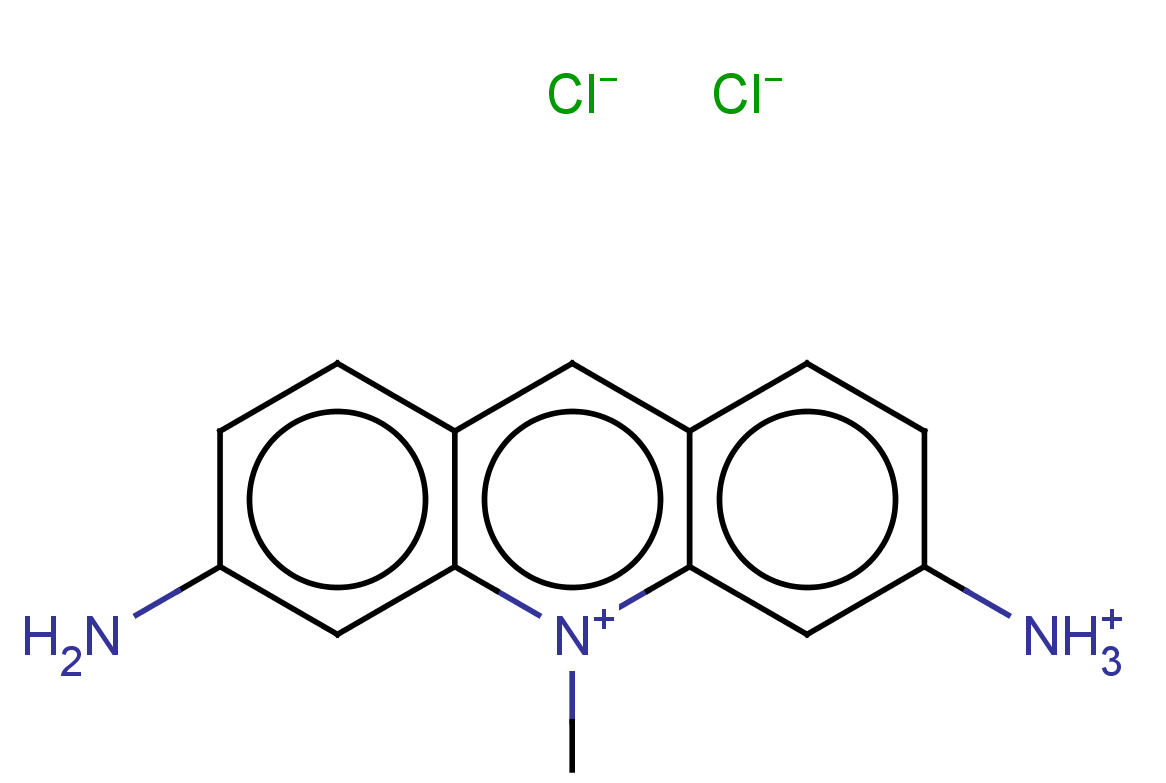 | miRNA | Inactive | Inactive |
| CID162282 [5] | 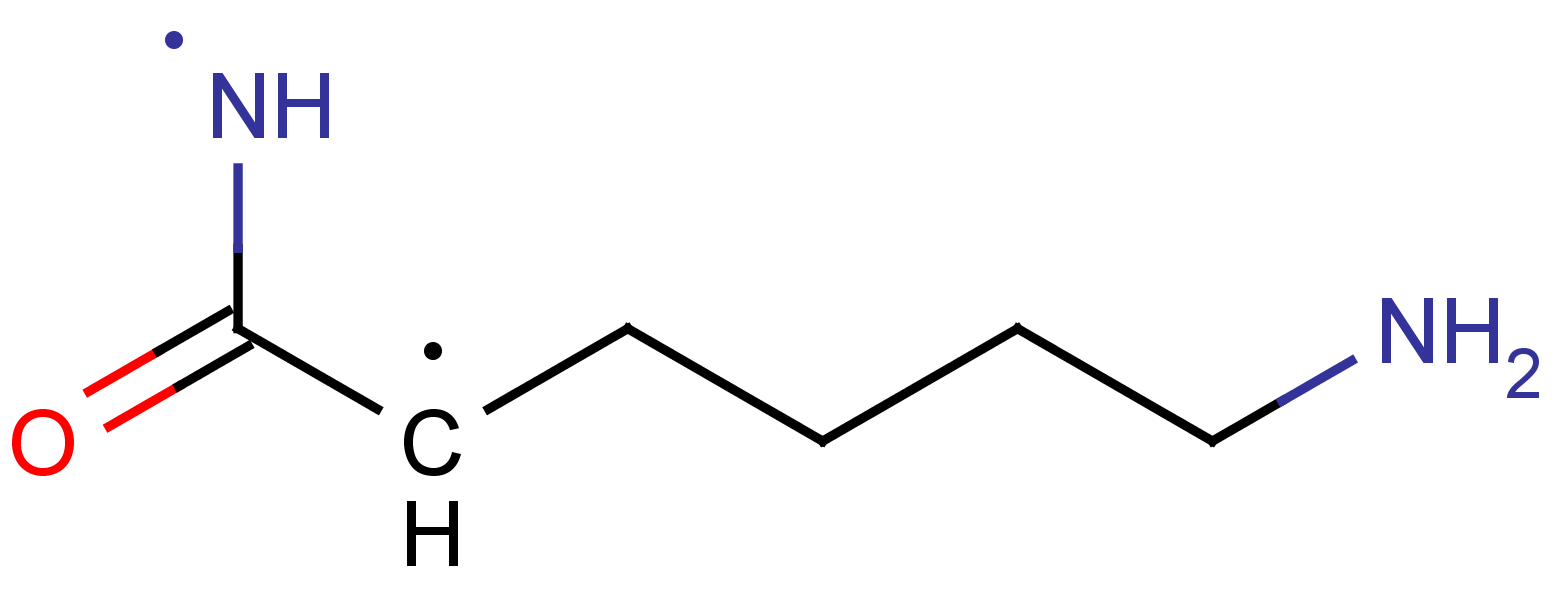 | miRNA | Inactive | Inactive |

References

1. Young DD, Connelly CM, Grohmann C, Deiters A: **Small molecule modifiers of microRNA miR-122 function for the treatment of hepatitis C virus infection and hepatocellular carcinoma.** *J Am Chem Soc* 2010, **132:** 7976-7981.

2. Tan GS, Chiu CH, Garchow BG, Metzler D, Diamond SL, Kiriakidou M: **Small molecule inhibition of RISC loading.** *ACS Chem Biol* 2012, **7:** 403-410.

3. Melo S, Villanueva A, Moutinho C, Davalos V, Spizzo R, Ivan C *et al*.: **Small molecule enoxacin is a cancer-specific growth inhibitor that acts by enhancing TAR RNA-binding protein 2-mediated microRNA processing.** *Proc Natl Acad Sci U S A* 2011, **108:** 4394-4399.

4. Bose D, Jayaraj G, Suryawanshi H, Agarwala P, Pore SK, Banerjee R *et al*.: **The tuberculosis drug streptomycin as a potential cancer therapeutic: inhibition of miR-21 function by directly targeting its precursor.** *Angew Chem Int Ed Engl* 2012, **51:** 1019-1023.

5. Watashi K, Yeung ML, Starost MF, Hosmane RS, Jeang KT: **Identification of small molecules that suppress microRNA function and reverse tumorigenesis.** *J Biol Chem* 2010, **285:** 24707-24716.
